# Formate as electron carrier in the gut acetogen *Blautia luti*: a model for electron transfer in the gut microbiome

**DOI:** 10.1080/19490976.2025.2609406

**Published:** 2026-01-02

**Authors:** Raphael Trischler, Volker Müller

**Affiliations:** a Molecular Microbiology & Bioenergetics, Institute of Molecular Biosciences, Johann Wolfgang Goethe University, Frankfurt, Germany

**Keywords:** Microbiome, *Blautia*, formate, hydrogen, cross-feeding, gut acetogens, Wood-Ljungdahl pathway, succinate, electron carrier, pyruvate-formate-lyase

## Abstract

Species of the genus *Blautia* are commonly found in the human gut and are known to be beneficial for the human well-being. However, only little is known about the physiology and the specific role of *Blautia* species in the human gut. In this study, we investigated the heterotrophic metabolism of the formate dehydrogenase lacking gut acetogen *Blautia luti*. We identified acetate, succinate, lactate, formate, and hydrogen as end products of sugar fermentation. Interestingly, formate is produced by the pyruvate-formate lyase reaction and used as electron acceptor in the Wood–Ljungdahl pathway of CO_2_ fixation. Thus, formate connects the oxidative branch of glucose metabolism with the reductive branch. The use of formate as an intraspecies electron carrier seems to be common in gut acetogens. This study highlights the role of formate as electron carrier in the gut microbiome and improves our understanding of the physiology of *Blautia* species in the human gut. It also introduces *B. luti* as potential candidate for biotechnological applications due to the production of highly desired succinate.

## Introduction

The human gut is a highly complex ecosystem that consists of trillions of microorganisms.^1^ This community is made of mainly bacteria, but also archaea, viruses, and eukaryotic cells.^2^ The composition of the microbiome and the presence of specific microbes in the gut is not only important for the breakdown of organic matter, it also impacts human health as well as the development of diseases. Since many different factors such as diet, pharmaceuticals, geography, stress, lifestyle, and many more shape the gut microbiome, each human being is the architect of its own gut microbiome.^3-6^ One taxon of gut bacteria that typically correlates to human well-being is the genus *Blautia,* especially due to the production of short-chained fatty acids and succinate, its biotransformation and antibacterial activity.^7^^,^^8^

Species of the genus *Blautia* typically belong to the nonphylogenetic group of acetogenic bacteria, which are defined as strictly anaerobic bacteria that can reduce two mol of CO_2_ by the Wood–Ljungdahl pathway (WLP) to acetyl-CoA. Most acetogens produce acetate from acetyl-CoA *via* acetyl phosphate, since this reaction results in the production of ATP.^9-12^ The WLP is divided into two branches: The methyl-branch and the carbonyl-branch. In the methyl-branch, CO_2_ is first reduced to formate which is then subsequently further reduced to a methyl group. In the carbonyl-branch, CO_2_ is reduced to enzyme-bound CO, which is then condensed with the methyl group and CoA to acetyl-CoA. This reaction and the production of CO are catalyzed by the bifunctional CO dehydrogenase/acetyl-CoA synthase complex (CODH/ACS).^13-15^ The CODH/ACS functions as a key enzyme of the WLP, since it combines the two WLP branches and catalyzes the reaction in the WLP with the highest thermodynamic barrier, the reduction of CO_2_ to CO.^16^ This reaction can only be catalyzed with reduced ferredoxin as an electron donor due to the negative redox potential of CO_2_. In general, acetogens can grow autotrophically on H_2_ and CO_2_ by utilization of the WLP, which results in the production of acetate as end product.^17^^,^^18^ Under this condition H_2_ functions as electron donor for the reduction of electron carriers used by the WLP. However, the redox potential of H_2_ (E_0_′ = −414 mV)^19^ is too low for reduction of ferredoxin (E_0_′ = −400 to −500 mV).^16^^,^^20^ To overcome this barrier, acetogens utilize the mechanism of electron bifurcation.^21^^,^^22^ The electron-bifurcating hydrogenase utilizes H_2_ as electron donor for the exergonic reduction of NADH. The energy of this reaction is used to drive the endergonic reduction of ferredoxin.^23^^,^^24^ The electron carriers used in the methyl-branch can differ between acetogens. Therefore, many acetogens harbor an electron-bifurcating Nfn or Stn complex to convert NADH and ferredoxin to NADPH.^25^^,^^26^ The reduction of CO_2_ to acetate by the WLP results in a net ATP formation of zero, since one ATP is consumed in the methyl-branch and one ATP is produced by the acetate kinase reaction.^10^ An additional energy-conserving mechanism has to be used for acetogenesis from H_2_ + CO_2_. To date, two respiratory enzyme complexes have been found in acetogens, which produce an ion/proton gradient across the membrane as fuel for ATP synthase for ATP production, the ferredoxin:NAD^+^ oxidoreductase (Rnf) and the ferredoxin:H^+^ oxidoreductase (Ech) complex.^16^

Acetogens are not limited to the utilization of H_2_ + CO_2_ as growth substrates, they can use various substrates for organoheterotrophic growth such as different sugars, alcohols, carboxylic acids, aldehydes, amino acids or methylated compounds.^9^^,^^16^^,^^27-37^ The flexibility in the usage of different growth substrates gives acetogens an enormous ecological advantage, which can be of great importance for interspecies interactions in the human gut. In addition, the oxidation of carbohydrates is directly linked to the WLP as electron sink. During this process, the respiratory enzymes (Rnf or Ech) lead to additional ATP production, resulting in an energetic advantage of acetogens in contrast to other fermenters.^10^^,^^16^

Recently, *Blautia* strains with an unusual WLP were identified.^38^ The genome of these *Blautia* strains does encode for all WLP enzymes except for a formate dehydrogenase, which catalyzes the reduction of CO_2_ to formate as the first reaction of the methyl-branch in the WLP. In addition, it was experimentally proven that cells of the formate dehydrogenase lacking *Blautia* group were able to perform acetogenesis from CO and formate, demonstrating an unusual but active WLP even in the absence of a formate dehydrogenase.^38^ One of these strains is the gut acetogen *Blautia luti*. However, little is known about the physiology of this bacterium and how the WLP interacts with the oxidative branch of its metabolism. This study revealed that *B. luti* produces acetate, succinate, lactate, formate, and H_2_ as end products during fermentation of various carbohydrates. Formate is produced during heterotrophic metabolism by pyruvate-formate lyase and can subsequently be used as an electron acceptor in the methyl-branch of the WLP instead of CO_2_. This feature of gut acetogens helps to further understand the interactions of different gut bacteria and their role in human well-being. In addition, this study is of interest for biotechnological applications since it also introduces *B. luti* as a bacterium producing the industrial desired compound succinate.

## Material and methods

### Organism and cultivation

*B. luti* DSM14534 was cultivated at 37 °C in CO_2_/KHCO_3_-buffered complex medium.^39^ For cultivation under CO_2_/KHCO_3_-limited conditions medium was used as described.^40^ As carbon and energy source 20 mM of glucose, xylose, arabinose, sorbitol, sucrose, trehalose, maltose, or raffinose were used. In addition, some cultures grew in presence of 75 mM Na^+^-phosphinate. Growth was monitored by measuring the optical density at 600 nm (OD_600_).

### Preparation of resting cells

*B. luti* DSM14534 was grown in CO_2_/KHCO_3_-buffered complex medium with 20 mM glucose as carbon and energy source to late exponential growth phase. Resting cells were prepared as described.^38^ The protein concentration was measured as described.^41^

### Cell suspension experiments

Cell suspension experiments were carried out at 37 °C in 115-ml serum flasks, which contained 10 ml imidazole buffer (50 mM imidazole, 20 mM KCl, 20 mM NaCl, 20 mM MgSO_4_, 2 mM DTE, 4.4 µM resazurin, pH 7.0) with 60 mM KHCO_3_ under a N_2_/CO_2_ [80:20 (v:v)] atmosphere or in buffer without KHCO_3_ under a N_2_ atmosphere. Resting cells were resuspended to a total protein concentration of 1 mg/ml. In each experiment, glucose (10 mM) was used as carbon source. For the mixotrophic fermentation experiments 1 bar 100% H_2_ + CO_2_ [80:20 (v/v)] or 20% of CO were added to the atmosphere.

### Determination of metabolites

The concentrations of the metabolites acetate, formate, lactate, and succinate as well as the concentrations of different sugars (glucose, xylose, arabinose, sucrose, trehalose, maltose, raffinose, and sorbitol) were determined by high-performance liquid chromatography as described.^34^^,^^40^ The concentrations of H_2_^42^ and CO^43^ were measured by gas chromatography as described.

### Preparation of cell-free extract

For preparation of cell-free extract *B. luti* was cultivated with 20 mM glucose, sorbitol or maltose to mid exponential growth phase (OD_600_ = 2). Cell-free extract was prepared as described.^38^ The protein concentration was measured according to Bradford.^44^

### Enzyme assays

The determination of enzyme activities in cell-free extract was performed under strictly anoxic conditions at 37 °C in glass cuvettes (d = 0.2  cm; Glasgerätebau Ochs, Germany) in Tris-buffer (100 mM Tris-HCl, 2 mM DTE, 4.4 µM resazurin, pH 7.5). For measurement of lactate dehydrogenase activity 0.25 mM NADH were used as electron donor and 20 mM pyruvate as electron acceptor. The oxidation of NADH was determined at 340 nm. The determination of malate dehydrogenase activity was performed similar to the lactate dehydrogenase assay, but with 5 mM oxaloacetate as electron acceptor. For fumarate reductase activity 10 mM methylviologen, previously reduced by addition of sodium dithionite, were used for reduction of 10 mM fumarate. The oxidation of methylviologen was measured at 604 nm. To determine pyruvate formate lyase activity in Tris-buffer (100 mM Tris-HCl, 20 mM MgSO_4_, pH 7.5, 2 mM DTE, 4.4 µM resazurin, pH 7.5) formate production from 10 mM pyruvate in presence of 1.75 mM CoA was measured over time using the “formic acid assay kit” from R-Biopharm AG (Darmstadt, Germany). Pyruvate ferredoxin oxidoreductase was measured in the same buffer in presence of 100 µM thiaminpyrophosphate and 200 µM CoA by the reduction of 3 mM ferredoxin with 10 mM pyruvate as electron donor. The reaction was monitored at 430 nm.

### Analysis of transcript abundance by semiquantitative PCR

*B. luti* was cultivated with 20 mM glucose as a carbon and energy source to mid exponential growth phase. The isolation of RNA, digestion of chromosomal DNA and synthesis of cDNA was performed as described.^29^ The cDNA was used as template for determination of transcript abundance by semiquantitative PCR as described.^45^ The primers used in this study are listed in Supplementary Table S1.

## Results

### Sugar fermentation by *B. luti*

**Figure 1.**
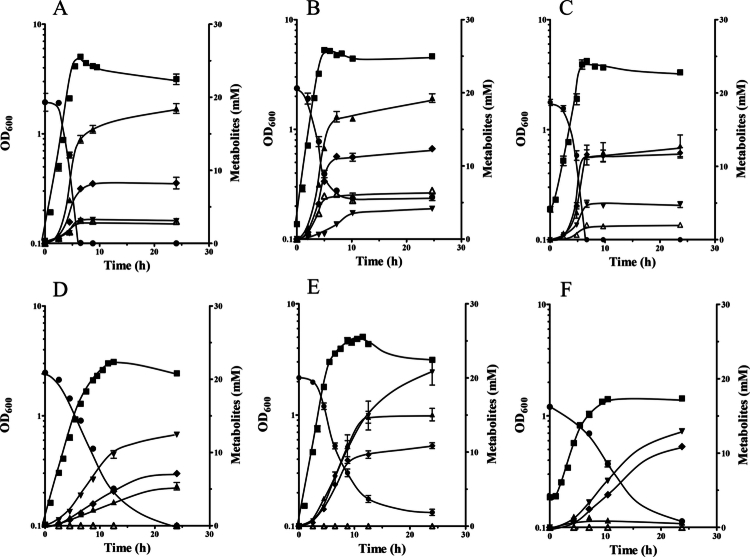
Growth of *B. luti* with glucose (A, D), maltose (B, E), and sorbitol (C, F) as carbon and energy source in absence (A-C) or presence (D-F) of Na^+^-phosphinate. *B. luti* was grown at 37 °C in 115 ml serum flasks containing 50 ml CO_2_/KHCO_3_-buffered medium with 20 mM of glucose (A), maltose (B) or sorbitol (C) as substrate. In addition, *B. luti* was also grown in presence of 75 mM Na^+^-phosphinate with 20 mM of glucose (D), maltose (E), and sorbitol (F) as substrate. For inoculation (**5%**) a preculture without Na^+^-phosphinate was used that was previously adapted to the corresponding sugar. The optical densities at 600 mM (■), as well as the concentrations of each sugar (●), acetate (▲), succinate (♦), formate (Δ), and lactate (▼) were determined. Each data point are mean ± SEM; *N* = 3 independent experiments.

*B. luti* is known to grow heterotrophically on many different sugars;^46^ however, growth and fermentation profiles have never been reported in the literature. When grown in CO_2_/KHCO_3_-buffered medium with 20 mM glucose as carbon and energy source, *B. luti* grew with a growth rate of 0.69 ± 0.01 h^−1^ to a maximal optical density of 5.05 ± 0.13 (Figure 1A). Glucose was completely consumed at the end of fermentation, the major products were acetate (18.46 ± 1.35 mM), succinate (8.26 ± 1.58 mM), lactate (3.05 ± 0.47 mM), and formate (3.01 ± 0.21 mM). Furthermore, small amounts of H_2_ were measured at the end of fermentation (Table 1). The fermentation balance was as follows:

(1) $$1\;\mathrm{glucose}\to 0.97\;\mathrm{acetate}+0.42\;\mathrm{succinate}+0.15\;\mathrm{lactate}+0.15\;\mathrm{formate}+0.17\;H_{2}$$

**Table 1.** Fermentation balance and growth parameters of *B. luti* grown on different carbohydrates as carbon and energy source.

	Xylose C_5_H_10_O_5_	Arabinose C_5_H_10_O_5_	Glucose C_6_H_12_O_6_	Sorbitol C_6_H_14_O_6_	Sucrose C_12_H_22_O_11_	Trehalose C_12_H_22_O_11_	Maltose C_12_H_22_O_11_	Raffinose C_18_H_32_O_16_	
Fermentation balance (metabolite/carbohydrate)									
Acetate	1.39 ± 0.09	1.09 ± 0.03	0.97 ± 0.20	0.67 ± 0.18	1.50 ± 0.09	1.57 ± 0.21	1.29 ± 0.04	2.08 ± 0.3	
Succinate	0.26 ± 0.01	0.29 ± 0.01	0.42 ± 0.04	0.62 ± 0.10	0.79 ± 0.02	0.96 ± 0.16	0.83 ± 0.01	1.11 ± 0.12	
Lactate	0.05 ± 0.01	0.02 ± 0.01	0.16 ± 0.01	0.25 ± 0.04	0.32 ± 0.01	0.02 ± 0.01	0.28 ± 0.01	0.49 ± 0.07	
Formate	0.09 ± 0.02	0.28 ± 0.01	0.15 ± 0.01	0.10 ± 0.02	0.31 ± 0.02	0.72 ± 0.16	0.45 ± 0.01	0.38 ± 0.05	
H_2_	0.19 ± 0.04	0.26 ± 0.04	0.17 ± 0.03	0.29 ± 0.19	0.95 ± 0.20	0.68 ± 0.18	0.63 ± 0.08	1.29 ± 0.14	
Carbon balance									
C_Acetate_ (%)	55.4 ± 3.5	43.4 ± 1.4	32.5 ± 6.8	22.2 ± 5.9	25.1 ± 1.5	26.2 ± 3.4	21.6 ± 0.7	23.1 ± 3.3	
C_Succinate_ (%)	21.1 ± 0.8	23.2 ± 0.6	28.3 ± 2.7	41.5 ± 7.0	26.3 ± 0.5	32.0 ± 5.5	27.7 ± 0.4	24.6 ± 2.6	
C_Lactate_ (%)	2.7 ± 0.8	1.5 ± 0.1	7.9 ± 0.3	12.5 ± 2.2	8.1 ± 0.2	0.4 ± 0.1	7.0 ± 0.4	8.2 ± 1.2	
C_Formate_ (%)	1.9 ± 0.4	5.6 ± 0.1	2.5 ± 0.2	1.7 ± 0.4	2.6 ± 0.1	6.0 ± 1.4	3.7 ± 0.1	2.1 ± 0.3	
Growth parameters									
Growth rate (h^−1^)	0.64 ± 0.01	0.65 ± 0.01	0.67 ± 0.01	0.59 ± 0.05	0.64 ± 0.01	0.58 ± 0.02	0.77 ± 0.03	0.77 ± 0.03	
Final OD_600_	3.59 ± 0.69	3.34 ± 0.47	5.05 ± 0.13	4.37 ± 0.36	7.33 ± 0.17	2.99 ± 0.04	5.29 ± 0.21	6.52 ± 0.70	

Noteworthy, *B. luti* was not able to grow in the absence of CO_2_/KHCO_3_ (data not shown). When grown with different carbon sources such as xylose, arabinose, sucrose, trehalose, maltose or raffinose *B. luti* always produced acetate as major end product, followed by succinate, lactate, and formate (Figure 1B and C; Supplementary Figure S1; Table 1). In addition, H_2_ was produced as fermentation end product from every sugar tested. Interestingly, growth on the sugar alcohol sorbitol led to production of acetate and succinate in almost equal amounts, resulting in the highest succinate production per hexose molecule (Table 1). The fermentation balance for growth of *B. luti* on sorbitol as carbon and energy source was as follows:

(2) $$1\;\mathrm{sorbitol}\to 0.67\;\mathrm{acetate}\;+\;0.62\;\mathrm{succinate}\;+\;0.25\;\mathrm{lactate}\;+\;0.10\;\mathrm{formate}\;+\;0.29\;H_{2}$$

Obviously, succinate was formed instead of acetate.

### Formate as a central metabolite in *Blautia* species lacking formate dehydrogenases

The genome of *B. luti* does not harbor a formate dehydrogenase-encoding gene,^38^ but formate can be fed into the WLP directly. Therefore, the question arises of how formate is produced during growth on all the sugars tested. Analysis of the genome of *B. luti* revealed the presence of two copies of a pyruvate-formate lyase (PFL)-encoding gene (Supplementary Table S2), which we suspected to catalyze formate formation. To test this hypothesis, growth on glucose in the presence of the PFL inhibitor Na^+^-phosphinate was investigated (Figure 1D). The growth rate was reduced to 0.38 ± 0.01 h^−1,^ and the fermentation profile changed dramatically. Formate was no longer produced. Furthermore, acetate (5.39 ± 0.88 mM) was produced in reduced amounts, and lactate was the major fermentation end product (12.43 ± 0.47 mM). Succinate production was only slightly decreased. The fermentation balance of glucose in the presence of Na^+^-phosphinate was as follows:

(3) $$1\;\mathrm{glucose}\to \;0.26\;\mathrm{acetate}\;+\;0.34\;\mathrm{succinate}\;+\;0.60\;\mathrm{lactate}\;+\;0.00\;\mathrm{formate}\;+\;0.11\;H_{2}$$

The same effect of Na^+^-phosphinate was also observed during fermentation of the disaccharide maltose (Figure 1E). In the absence of Na^+^-phosphinate, acetate (19.28 ± 1.01 mM) was the major fermentation end product, followed by succinate (12.34 ± 0.29 mM), H_2_ (9.45 ± 1.30 mM), formate (6.65 ± 0.38 mM), and lactate (4.15 ± 0.10 mM). However, in the presence of Na^+^-phosphinate, lactate was again the major fermentation product (20.80 ± 3.05  mM), followed by acetate (15.06 ± 1.45 mM), H_2_ (11.7 ± 0.7 mM), and succinate (10.86 ± 0.68 mM). Moreover, formate was not produced in the presence of Na^+^-phosphinate.

To test whether carbon flow can also be redirected to succinate production as an electron sink instead of the WLP or lactate, we tested growth of *B. luti* with sorbitol in the presence of Na^+^-phosphinate (Figure 1F), since this sugar led to the highest succinate production (see above). As expected, growth rate (0.38 ± 0.04 h^−1^), and final OD (1.48 ± 0.07) were highly reduced in comparison to growth without Na^+^-phosphinate (0.50 ± 0.05 h^−1^; final OD 3.60 ± 0.19). Surprisingly, while acetate was now only produced in trace amounts (0.67 ± 0.12 mM), succinate (10.87 ± 0.11), and lactate (12.94 ± 0.40 mM) were produced in almost equal amounts as the major fermentation end products. Unexpectedly, the amount of H_2_ was slightly reduced at the end of fermentation to 1.9 ± 0.1 mM. Additionally, the production of succinate was similar to Na^+^-phosphinate-free conditions, revealing that lactate production is the preferred electron sink in the absence of the WLP.

These results are in line with the hypothesis that PFL is used during heterotrophic growth for pyruvate oxidation. The most important observation is the absence of formate production during heterotrophic fermentation in presence of Na^+^-phosphinate, but also the increased lactate and decreased acetate production is in line with the use of PFL. We postulate, that formate, which is produced by PFL, is used by *B. luti* as electron acceptor in the WLP to produce additional acetate. As compensation for the loss of formate by inhibition of PFL, pyruvate is further metabolized to lactate as electron sink alternative to the WLP. However, since acetate production is not completely abolished, additional acetyl-CoA forming enzymes such as the pyruvate-ferredoxin-oxidoreductase (PFOR) must be active.

### Enzyme activities in cells grown under different conditions

**Figure 2.**
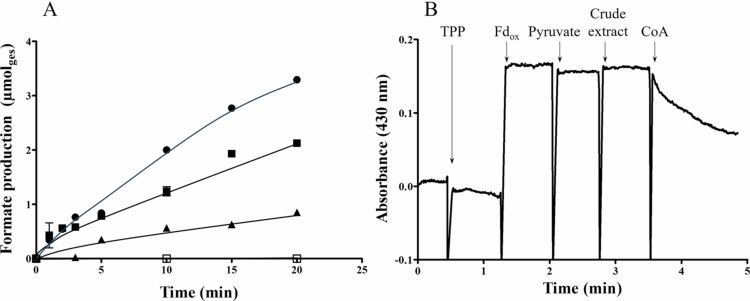
Measurement of PFL (A) and PFOR activity (B) in crude extract of *B. luti* grown on 20 mM glucose. (A) PFL activity was determined by formate production from 10 mM pyruvate and 1.75 mM CoA with 0.75 (●), 0.5 (■), and 0.25 mg ml^−1^ (▲) crude extract. In addition, formate production from pyruvate with 0.5 mg ml^−1^ (□) crude extract in absence of CoA was determined. Each data point are mean ± SEM; *N* = 2 independent experiments. (B) For measurement of PFOR activity the reduction of 3 mM ferredoxin with 10 mM pyruvate, 200 µM CoA, and 100 µM thiamine pyrophosphate (TPP) as substrates was monitored at 430 nm. Both reactions were performed in Tris-buffer (100 mM Tris-HCl, 20 mM MgSO_4_, pH 7.5, 2 mM DTE, 4.4 µM resazurin, pH 7.5) at 37 °C.

To verify, whether PFL or PFOR are both active during heterotrophic metabolism, we measured both activities in crude extract of *B. luti* grown on glucose (Figure 2, Table 2). As expected, crude extract of *B. luti* was able to produce formate form pyruvate in the presence of CoA with an activity of 292 ± 69 mU mg^−1^, confirming an active PFL (Figure 2A). Without addition of CoA, formate was not produced. Additionally, we measured PFOR activity. Indeed, crude extract of *B. luti* was also able to reduce ferredoxin with pyruvate as substrate in presence of CoA with an activity of 1019 ± 235 mU mg^−1^ (Figure 2B). Ferredoxin could not be reduced in the absence of CoA. Furthermore, we were able to measure a NADH-dependent lactate dehydrogenase activity (NADH:pyruvate oxidoreductase activity) of 209 ± 16 mU mg^−1^. These results reveal that pyruvate is metabolized in *B. luti via* three routes: First, it can be used as substrate by PFL for production of acetyl-CoA and formate. The latter can than be used by the WLP as electron acceptor but also for cross-feeding in the gut. Next, pyruvate can also be used by PFOR for production of acetyl-CoA and reduction of ferredoxin. Reduced ferredoxin can be used in the WLP but also as source for molecular hydrogen. And last, pyruvate can be reduced to lactate.

**Table 2.** Activities of enzymes in crude extract of *B. luti* grown in absence or presence of Na^+^-phosphinate with 20 mM glucose, sorbitol or maltose as carbon and energy source.

Enzyme activity	Substrates	Specific activity (mU mg^−1^)^a^ in cells grown on:					
		Glucose	Sorbitol	Maltose	Glucose + Phosphinate	Sorbitol + Phosphinate	Maltose + Phosphinate
PFOR	Fd_ox_ + Pyruvate + CoA + TPP	1162 ± 129	3648 ± 382	1452 ± 268	2300 ± 182	5882 ± 605	2106 ± 122
PFL	Pyruvate + CoA	292 ± 69	230 ± 13	575 ± 49	0.0	0.0	0.0
Lactate dehydrogenase	Pyruvate + NADH	209 ± 16	289 ± 54	324 ± 90	246 ± 18	235 ± 27	295 ± 28
Fumarate reductase	Fumarate + MV_red_	504 ± 53	880 ± 44	852 ± 22	682 ± 54	989 ± 15	695 ± 110
Malate dehydrogenase	Oxaloacetate + NADH	1070 ± 256	923 ± 141	668 ± 67	690 ± 193	2839 ± 246	496 ± 90
Hydrogenase	H_2_ + MV	290 ± 33	644 ± 37	19 ± 0.3	131 ± 9	492 ± 39	14 ± 3

a All activities were determined as described in “Experimental Procedures”. PFOR, pyruvate-ferredoxin oxidoreductase; PFL, pyruvate-formate lyase. The concentration of Na^+^-phosphinate was 75 mM. All values are mean ± SEM; N ≥ 5.

Interestingly, addition of Na^+^-phosphinate had stronger effects on the production of acetate during growth on sorbitol than on acetate production from glucose or maltose (see above), indicating that PFL or PFOR might be differentially produced during growth on these sugars. Unexpectedly, PFL activity was almost similar in crude extract of *B. luti* grown on all of these sugars (Table 2). However, PFOR activity was approximately three times increased in crude extract of cells previously grown on sorbitol (3648 ± 382 mU mg^−1^) in comparison to crude extract of glucose (1019 ± 235 mU mg^−1^) and maltose (1452 ± 268 mU mg^−1^) grown cells (Table 2). The NADH:pyruvate oxidoreductase activity was comparable in crude extract of glucose, maltose, and sorbitol grown cells (192 ± 22 mU mg^−1^; 323 ± 90 mU mg^−1^; 289 ± 54 mU mg^−1^). Due to inhibition of PFL by Na^+^-phosphinate pyruvate can only be metabolized *via* the production of lactate or the PFOR reaction. Since the oxidation of the sugar alcohol sorbitol already leads to production of more reducing equivalents than the oxidation of glucose, the abolishment of acetate production and switch to lactate production might be a protective mechanism against accumulation of reducing power.

In addition, crude extract of cells cultivated with glucose, sorbitol or maltose in presence of Na^+^-phosphinate showed no PFL activity, but PFOR activity was highly increased by approximately 98%, 61%, and 45%, respectively, in comparison to crude extract of cells cultivated in absence of Na^+^-phosphinate. Additionally, the cultivation in presence of Na^+^-phosphinate had no effect on lactate dehydrogenase activity. A comparable effect was observed by cultivation of the gut bacterium *Roseburia intestinalis*, which uses PFOR and PFL simultaneously during heterotrophic fermentation at high iron concentration, but switches from PFOR usage to lactate dehydrogenase utilization at low iron concentrations.^47^ Therefore, a switch between PFL, PFOR, and lactate dehydrogenase at different growth conditions might also be a common feature for gut acetogens.

To further reconstruct the pathway for the heterotrophic metabolism of *B. luti*, the route for succinate production was investigated next. The generation of succinate is possible *via* three metabolic routes: The ATP-generating carboxylation of phosphoenolpyruvate (PEP) to oxaloacetate by PEP carboxykinase, the carboxylation of pyruvate to oxaloacetate by pyruvate carboxykinase or the carboxylation of pyruvate to malate catalyzed by the decarboxylating malate dehydrogenase. Of these three, only the PEP carboxykinase and the decarboxylating malate dehydrogenase encoding genes are present in the genome of *B. luti* (Supplementary Table S2)*.* We were able to measure a NADH-dependent reduction of oxaloacetate to malate in crude extract of *B. luti* grown on glucose with an activity of 1070 ± 256 mU mg^−1^, which is in line with the hypothesis that oxaloacetate is an intermediate of glucose fermentation in *B. luti*. The MDH activity was comparable in crude extract of maltose- (668 ± 67 mU mg^−1^) and sorbitol- (923 ± 141 mU mg^−1^) grown cells. Malate is then further metabolized to fumarate and finally reduced to succinate. Crude extract reduced fumarate with reduced methylviologen as electron donor with an activity of 499 ± 79  mU mg^−1^. Interestingly, crude extract of *B. luti* grown on maltose or sorbitol showed an almost two times higher fumarate reductase activity (Table 2). Na^+^-phosphinate did not affect fumarate reductase activity. Interestingly, addition of Na^+^-phosphinate to cells cultivated with glucose led to a decrease in malate dehydrogenase activity while the activity was approximately three times higher in sorbitol-grown cells. This might indicate that in absence of a functional WLP, succinate production might be used as alternative electron sink during fermentation of sorbitol, second to lactate production. The H_2_:MV-oxidoreductase activity measured in all crude extracts tested, was relatively low (approximately 640 to 20 mU mg^−1^) in comparison to other acetogens like *Blautia schinkii* with approximately 23,000 mU mg^−1^.^38^ In crude extract of *B. luti* cultivated in presence of Na^+^-phosphinate, hydrogenase activity was reduced by approximately 30%–50%. These results are in line with the reduced H_2_ production of growing cells of *B. luti* in the presence of Na^+^-phosphinate (see above). The absence of formate production by PFL and increased PFOR activity due to the addition of Na^+^-phosphinate suggests increased production of reduced ferredoxin under these conditions. However, without formate the WLP cannot be used as electron sink. Therefore, an alternative electron sink is needed. Unexpectedly, lactate production seems to be the favored as alternative electron sink to the WLP instead of H_2_ production, which is commonly found in acetogens. The production of lactate might even be favored in terms of energetics, since the interconversion of reduced ferredoxin to NADH is performed *via* the energy-conserving Rnf reaction. In sum, the data demonstrates that PFL plays a central role in the heterotrophic metabolism of *Blautia* species lacking formate dehydrogenases such as *B. luti*.

### Formate as a transient intermediate during mixotrophic fermentation

**Figure 3.**
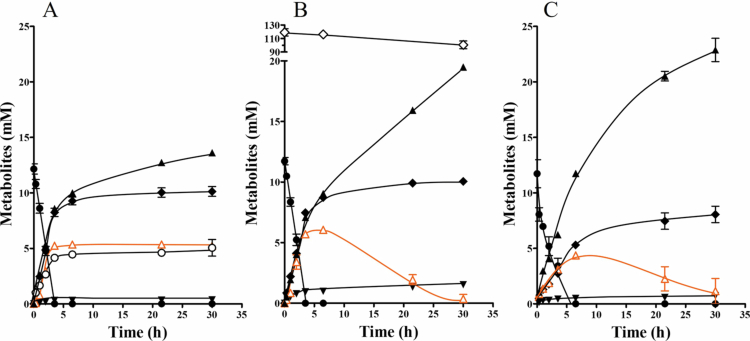
Fermentation of glucose in the presence of hydrogen or carbon dioxide by the resting cells of *B. luti*. *B. luti* was cultivated at 37 °C in CO_2_/KHCO_3_-buffered medium with 20 mM glucose as substrate to late exponential growth phase, harvested, washed, and resuspended in 10 ml imidazole buffer (50 mM imidazole, 60 mM KHCO_3_, 20 mM NaCl, 20 mM MgSO_4_, 2 mM DET, 4.4 µM resazurin, pH 7.0) to a final protein concentration of 1 mg ml^−1^ within 115 ml serum flasks. The resting cells were incubated under a N_2_/CO_2_ atmosphere [80%/20% (v/v)] with 10 mM glucose (A), with 10 mM glucose in the presence of 20% CO in the gas phase (B) and with 10 mM glucose and an atmosphere of 1 bar H_2_ + CO_2_ (C). At each timepoint, the concentrations of glucose (●), acetate (▲), succinate (♦), formate (Δ), lactate (▼), and H_2_ (○) were determined. Each data point is the mean ± SEM; *N* = 2 independent experiments.

To further investigate carbon flow during glucose fermentation in the absence of biomass production, resting cells experiments were performed. *B. luti* was grown in CO_2_/KHCO_3_-buffered medium with 20 mM glucose as carbon- and energy source. Cells were harvested, washed, and resuspended in imidazole buffer. In buffer containing CO_2_/KHCO_3_ cells fermented glucose (12.15 ± 0.66 mM) to 13.62 ± 0.33 mM acetate, 10.12 ± 0.63 mM succinate, 5.23 ± 0.24 mM formate and 5.05 ± 1.06 mM H_2_. Only trace amounts of lactate (0.36 ± 0.05 mM) were produced (Figure 3A). A carbon balance of 101,6% ± 0.6% and an electron balance of 94.5% ± 0.1% were calculated. The fermentation balance was as follows:

(4) $$1\;\mathrm{glucose}\to 1.12\;\mathrm{acetate}\;+\;0.83\;\mathrm{succinate}\;+\;0.03\;\mathrm{lactate}\;+\;0.43\;\mathrm{formate}\;+\;0.41\;H_{2}$$

Interestingly, resting cells of *B. luti* were not able to ferment glucose in the absence of CO_2_/KHCO_3_ (Supplementary Figure S2), similar to growing cells. CO_2_ seems to play an essential role during sugar fermentation by *B. luti*. To be more specific, CO_2_ might not only be essential for the use of the WLP but might also be required to produce succinate from phosphoenolpyruvate since that pathway requires a carboxylation reaction.

To further study the role of the WLP as well as the role of formate during heterotrophic metabolism, fermentation of glucose by resting cells of *B. luti* in the presence of external electron donors was investigated, which might be a more accurate reflection of the gut environment. Since *B. luti* can produce acetate from formate and CO *via* the WLP,^38^ glucose fermentation in the presence of similar CO concentrations (20% CO) used by Trischler et al.^38^ was investigated first (Figure 3B). As expected, at the end of fermentation, the acetate level increased to 19.49 ± 0.40 mM. In addition, during the first seven hours of fermentation, approximately 5 mM formate was produced, but afterwards, it was completely consumed. The addition of CO did not affect the production of succinate; however, lactate levels slightly increased to 1.54 ± 0.08 mM. In total, a consumption of 18.5 ± 0.5 mM CO was measured. H_2_ was not produced. These products contained 78.2% ± 2.3% of the carbon and 99.0% ± 3.3% of the electrons from the utilized glucose and CO. The fermentation balance was as follows:

(5) 1glucose+1.57CO→1.66acetate+0.86succinate+0.13lactate+0.00formate

When the cells were incubated with glucose under an atmosphere of 1 bar overpressure of H_2_ + CO_2_ (Figure 3C), the fermentation profile was similar to that of fermentation with additional CO. Not only was the production of acetate increased to 22.88 ± 1.48 mM, but formate was also first produced and afterwards almost completely consumed. The level of succinate and lactate were only slightly affected. The fermentation balance was as follows:

(6) 1glucose→1.96acetate+0.69succinate+0.06lactate+0.09formate

In the gut colon, bacteria most likely encounter mixotrophic conditions. We speculate that in FDH-lacking gut acetogens, formate serves as an intraspecies as well as interspecies electron carrier and that formate reduction *via* the WLP can be commonly used as an electron sink.

### Hydrogenases of *B. luti*

**Figure 4.**
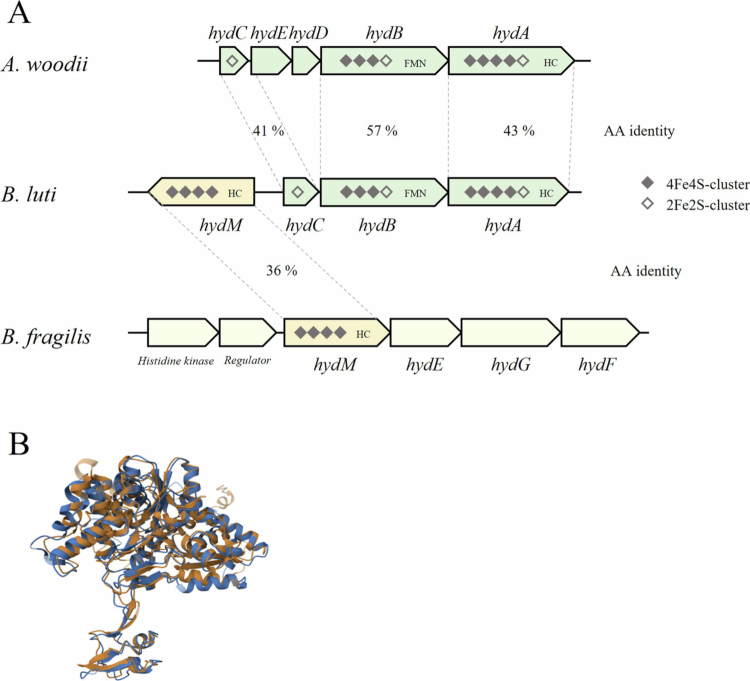
Comparison of the genetic organization and postulated cofactor composition of the hydrogenases of *B. luti*. (A) The genome of *B. luti* harbors a gene cluster consisting of genes encoding the electron-binding hydrogenase (*hydABC*) and group B [FeFe]-hydrogenase (*hydM*). The hydrogenase-encoding genes of *B. luti* were compared with the electron-bifurcating hydrogenase complex encoding genes (*hydABC*) of *A. woodii* as well as the postulated ferredoxin-dependent hydrogenase-encoding gene (*hydM*) of *B. fragilis*. (B) Comparison of the predicted structure of the HydM of *B. luti* (orange) and *B. fragilis* (blue). Structure predictions were performed by RCSB protein Data Bank.^48^

Since the resting cells of *B. luti* were able to utilize H_2_ as electron donor and growing cells produced H_2_ as fermentation end product, we further investigated the types of hydrogenases present in *B. luti*. Inspection of the genome sequence of *B. luti* revealed two hydrogenase-encoding genes *hydA* and *hydM*, which are located in close proximity to each other in the genome (Figure 4A). The gene *hydA* is part of a gene cluster with the genes *hydB* and *hydC*, similar to the electron-bifurcating hydrogenase-encoding gene cluster of *Acetobacterium woodii*. Comparison of the corresponding hydrogenase subunits revealed amino acid identities between 41% and 57%. In addition, the binding motifs for all cofactors of the *A. woodii* electron-binding hydrogenase complexes are conserved in the corresponding gene products of *B. luti* (Supplementary Figures S3, S4, S5).

In sharp contrast, *hydM* does not form a complex with other genes (Figure 4A). Further analysis of this gene revealed sequence motifs for four 4Fe-4S cluster and one H-cluster. The presence of a single hydrogenase subunit and composition of 4Fe-4S cluster led to the speculation that *hydM* encodes a group B [FeFe]-hydrogenase. Comparison of HydM to the group B [FeFe]-hydrogenase of *B. fragilis*^49^ lead to an amino acid identity of 36% (Figure 4A). However, on structural level both hydrogenases were almost identical (Figure 4B). Especially the typical formation of two distinct globular domains as well as the sequence motive for the H-cluster and four [4Fe4S] clusters are conserved. While the H-cluster and two [4Fe4S] clusters are present in the catalytic domain, the other two [4Fe4S] clusters are located in the smaller ferredoxin-like domain, which is typical for group B [FeFe]-hydrogenases. These hydrogenases are postulated to catalyze the production of H_2_ with reduced ferredoxin as an electron donor.^49^ Furthermore, we were able to identify the presence of HydM in several *Blautia* species, with amino acid identities between 71.9% and 99.8% (Supplementary Figures S6, S7).

### Reconstruction of the heterotrophic glucose fermentation pathway of *B. luti*

**Figure 5.**
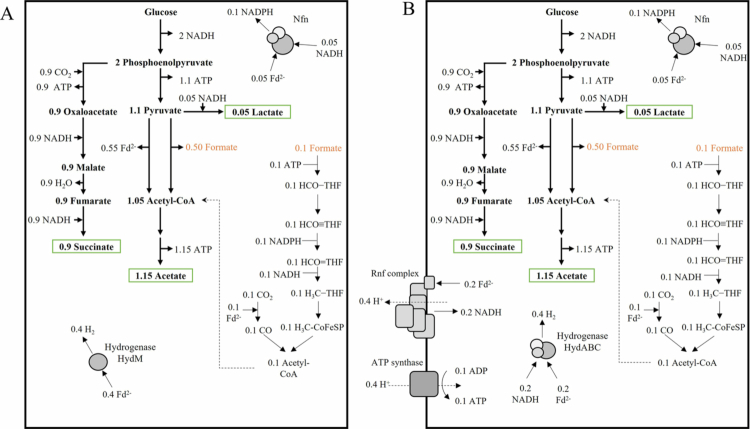
Biochemistry and bioenergetics of glucose fermentation by *B. luti*. (A) Glucose fermentation in the presence of HydM. (B) Glucose fermentation in the presence of the electron-bifurcating hydrogenase HydABC. For explanation, see text. Assumed stoichiometries: H^+^/ATP = 3.6 (ATP Synthase) and 2 H^+^/2e^−^ (Rnf).

Glucose is first metabolized by the Embden–Meyerhof–Parnas pathway (glycolysis). The intermediate phosphoenolpyruvate is further metabolized *via* oxaloacetate and malate to succinate. On the other hand, phosphoenolpyruvate is further metabolized to pyruvate. The latter can then be further metabolized to lactate or acetate by PFL or PFOR reactions as fermentation end products, as already discussed in detail. The WLP of *B. luti* uses a NADH-dependent methylene-THF reductase and an NADPH-dependent methylene-THF dehydrogenase.^38^ For NADP^+^ reduction and redox balancing, *B. luti* harbors genes encoding an electron-bifurcating Nfn complex. Furthermore, reduced ferredoxin, which is gained during glucose fermentation by PFOR reaction, is converted to NADH by the energy-conserving Rnf complex. This reaction leads to the establishment of a proton gradient across the membrane, which is used by ATP synthase for ATP production. To produce H_2_, two different hydrogenases can be used (see above). The postulated Fd-dependent HydM is commonly found in gut bacteria (Bacteroidetes and Firmicutes). However, the use of reduced ferredoxin as a direct electron donor for H_2_ production is a waste of energy for *B. luti*, since the use of ferredoxin by Rnf leads to energy conservation. A theoretical glucose fermentation pathway of *B. luti* that uses the Fd-dependent hydrogenase HydM and a NADH-dependent fumarate reductase would work independently of Rnf (Figure 5A). The calculated net ATP production would be 3.05 ATP/glucose. By use of the electron-bifurcating hydrogenase HydABC, only half the amount of ferredoxin would be used for hydrogen production. As a result, energy conversion by the Rnf would be possible (Figure 5B). Under these conditions, a net ATP production of 3.15 ATP/glucose can be calculated.

To tackle the question which hydrogenase is used by *B. luti* during growth on glucose, we analyzed transcript abundance of *hydA* and *hydM* with semiquantitative polymerase chain reaction (PCR) (Supplementary Figure S8). As a control, *frdAB*, encoding the fumarate reductase was amplified. *frdAB* was highly transcribed, which is in line with the production of succinate during growth on glucose and the enzyme measurements and reflects the high fumarate reductase activity measured in crude extract (see above). In case of the hydrogenase encoding genes, *hydA* was not expressed, but *hydM,* although expression levels were comparably low, which reflects the relatively low H_2_:MV oxidoreductase activity of crude extract of *B. luti* (Table 2).

## Discussion

*B. luti* is an acetogenic bacterium^38^ and found to be one of the most abundant of all *Blautia* species in the human gut.^38^^,^^50^ Acetogens are characterized by the WLP,^9^^,^^51^^,^^52^ a pathway that can convert C1-compounds at different redox levels. Reductants used are molecular hydrogen or carbon monoxide. Formate is an intermediate of the WLP and therefore, many acetogens can grow on external formate. In the absence of external reductants, three quarters of the formate have to be oxidized to CO_2_ to gain the electrons to reduce the other 25% to methyl-THF.^53^^,^^54^ In the presence of an external electron donor formate is exclusively reduced to acetate. This is what we observed in our studies.

The WLP also brings an ecological advantage to acetogens: disposal of electrons from sugar oxidation with CO_2_ or formate or a methyl-group (plus CO_2_) as electron acceptor. This allows homoacetogens the complete oxidation of the hexose (by glycolysis and pyruvate oxidation) to acetate which gives the highest ATP yield possible: 4 mol of ATP per mol of hexose by substrate-level phosphorylation.^10^^,^^55^ Acetogens have an oxidative and a reductive branch in their metabolism that are connect by various electron carriers, including H_2_. Sugars are oxidized and the reduced electron carriers are used in anaerobic respiration to reduce CO_2_, formate (+CO_2_) or methyl-groups (+CO_2_) *via* the WLP to acetate.^56^ What is described here for a single organism is also found between two different organism and a classical limb in an anaerobic food web. A strict fermenter oxidizes a hexose to 2 mol of acetate, 2 mol of CO_2_, and 4 mol of H_2_, which functions as electron carrier between two organisms. The oxidation of hydrogen by methanogens, sulfate reducers or acetogens lowers the hydrogen partial pressure in the environment and allows thermodynamically unfavorable hydrogen production by the fermenter and allows the hydrogen oxidizer to make a living as well.^57^ However, the free energy change decreases in the order of sulfidogenesis, methanogenesis and acetogenesis, and thus, methanogens and sulfate reducers outcompete acetogens on H_2_ utilization.^19^^,^^58^ Nevertheless, acetogens such as species of the genus *Blautia* can utilize an enormous range of carbohydrates and many other substrates (see above). Therefore, acetogens do not necessarily have to compete with methanogens or sulfate reducers for hydrogen to thrive in the gut. As shown here, the gut representative *B. luti* can grow on a number of different sugars that are oxidized *via* pyruvate as an intermediate, and electrons are channeled to the WLP but also to the production of reduced compounds such as succinate, lactate or H_2_. Pyruvate is oxidized by two different enzymes: PFL and PFOR. Whereas the former produces acetyl-CoA and formate, the latter produces acetyl-CoA and reduces ferredoxin and CO_2_. This makes a huge difference for the bacterium: formate can be directly used in the methyl-branch of the WLP as an electron acceptor, and NADH (from glycolysis) can be used directly or indirectly (converted to NADPH or reduced to ferredoxin by transhydrogenases) as an electron donor. From the data presented herein, it is clear that PFL is the major source of formate in *B. luti* and not formate dehydrogenase, which is lacking in *B. luti*. In the presence of CO or hydrogen, formate is only transiently produced from sugars and then completely reduced *via* the WLP to acetate. Like hydrogen, formate can also be used as an electron carrier between different species. The gut acetogen *Clostridium bovifaecis* also lacks a formate dehydrogenase but uses solely PFOR for pyruvate oxidation instead of PFL,^59^ in contrast to *B. luti*. Thus, formate must be provided by an external source, such as another bacterium, otherwise *C. bovifaecis* can no longer grow on sugars. The same effect of formate-dependent growth was observed by the genetic deletion of formate dehydrogenase/hydrogen-dependent CO_2_ reductase in the homoacetogens *T. kivui* or *A. woodii.*^60^^,^^61^ In sharp contrast, the production of formate by PFL enables *B. luti* to use the WLP as a reductive branch independent of formate cross-feeding. Interestingly, PFOR in addition to PFL is also present in *B. luti*, to produce reduced ferredoxin. This electron carrier could be reoxidized by the Rnf complex to reduce NAD^+^ and conserve additional energy or could be reoxidized by a hydrogenase (either by HydM or in combination with NADH by the electron bifurcating hydrogenase HydABC) to produce molecular hydrogen. The use of the postulated Fd-dependent hydrogenase HydM by *B. luti*, as our data suggest, is unexpected, since the use of the electron-bifurcating hydrogenase HydABC would provide an energetic advantage. However, the use of HydM is in line with the study of Welsh et al.^49^ which proposed that HydM is generally responsible for most H_2_ production in the human gut. Nevertheless, hydrogen production is only possible at low partial hydrogen concentrations since the redox potentials of ferredoxin (E_0_′ = −400 to −500 mV) and H_2_/H^+^ (E_0_′ = −414 mV) are very close.^16^^,^^19^ In addition, HydM could work as an emergency valve when the cytoplasm is too reduced. When the PFL was inhibited by phosphinate, PFOR did not step in. Instead, electrons were redirected from pyruvate to yield lactate as a major fermentation end product. Lactate formation under high electron loading was also observed in *A. woodii* and when the methylene-THF reductase was genetically deleted, *A. woodii* switched from acetogenesis to mixed acid fermentation.^61^^,^^62^

The fact that *B. luti* produces only minor amounts of hydrogen but can also use hydrogen as an electron donor might contribute to the finding that *Blautia* species are generally indicators of a healthy gut. Hydrogen is a central fermentation end product of carbohydrate breakdown in the gut microbiome. Its presence in the gut has positive and negative effects on human well-being. High hydrogen concentrations can impair the metabolism of gut bacteria,^19^ as already mentioned above and is connected to diseases such as carbohydrate malabsorption and inflammatory bowel disease.^63-65^ On the other hand, hydrogen can reduce oxidative stress and has positive effects on diseases such as obesity, inflammation, cancer or even Parkinson's disease.^66-68^ The finding that gut acetogens like *B. luti* can produce hydrogen and formate during carbohydrate fermentation but are also able to consume these compounds make gut acetogens appear in a new light. The primary role for acetogens might be the breakdown of carbohydrates to produce short-chain fatty acids, such as acetate, propionate, and butyrate, as well as lactate, succinate, formate, hydrogen and other compounds. These products might not only be directly useful for the gut epithel cells, but the fermentation end products can also be used for cross-feeding by other gut bacteria. In addition, acetogens can step in as hydrogen consumer to prevent accumulation of hydrogen in the gut, at times when methanogens or sulfate-reducing bacteria are not able to do so. In the rumen of sheep, for example, over 90% of the hydrogen is consumed by methanogens, the H_2_-utilization of acetogens is almost irrelevant. However, in the gut of methanogen-free sheep, acetogens were able to capture up to 25% of the hydrogen.^69^ Moreover, our study suggests that species of the genus *Blautia* might not only be able to prevent accumulation of H_2_ in the gut but also the accumulation of toxic amounts CO. Interestingly, CO is naturally produced by the human body in low concentrations (0.5 to 4.5 ppm CO each hour) mainly as result of heme degradation *via* heme oxygenase reaction.^70^^,^^71^ In low concentrations CO functions as anti-inflammatory agent,^72^ neurotransmitter,^73^^,^^74^ regulator of the mucosal immune response^75^ and has in general beneficial effects on the human well-being.^71^ However, due to external exposure to small amounts of CO over a longer period or due to exposure of higher concentrations in general (approximately >100 ppm), CO can also become a toxic compound due to binding and inhibition of haemoproteins.^76^^,^^77^ Interestingly, a CO concentration of 10 to 13 ppm could be measured in the flatulence of heathy people, while the CO concentration of people with gastrointestinal disorders was highly increased to 258 ppm,^78^ showing that the human gut microbiome contributes to CO homeostasis in the gut. We postulate that gut acetogens, especially species of the genus *Blautia* play a crucial role in CO uptake. In a recently published study,^38^ we were able to demonstrate CO utilization by the gut acetogens *B. wexlerae* and *B. luti*, that was further characterized in this study. Species of the genus *Blautia* devoid of a FDH can utilize CO in combination with formate to produce the short chained fatty acid acetate. These findings are further supported by the study of Katayama et al.^79^ which revealed that most CO dehydrogenase-encoding transcripts of the human gut microbiome belonged to species of the genus *Blautia*.

Another important feature of *B. luti* is the production of succinate as fermentation end product. In the human gut, succinate plays a major role in the maintenance of the gut homeostasis.^80^ On one site, the presence of succinate has many beneficial effects for human health. Succinate can help to prevent diseases such as obesity but also stimulates the host mucosal immune cells and the function of other gut microbes which leads to a healthy balance between gut bacteria and their host.^81^ Additionally, it can be directly used as energy source for gut epithelial cells, but can also be used by other gut microbes to produce healthy short-chain fatty acids.^80^^,^^82^ In a healthy gut, the succinate concentration is approximately 0.5 to 5 mM.^83^^,^^84^ However, gut dysbiosis is often accompanied by drastically increased succinate concentrations that can reach up to 25 mM.^85^ Many studies revealed that an abnormal increase of succinate levels in the gut is often accompanied with immune disorders and diseases such as inflammation and even cancer.^85-87^ Therefore, a balance between succinate producing bacteria such as *B. luti* and succinate fermenting bacteria is essential for a healthy gut.

Furthermore, succinate is a highly desirable product for biotechnology. In fact, succinate and other four carbon 1,4-diacids (fumarate and malate) are placed as number one of the 12 most valuable chemicals derived from sugars and synthesis gas.^88^ The biotechnologically relevant organisms such as *Actinocacillus succinogenes*, *Anaerobiospirillium succiniproducens*, *Mannheimia succiniproducens*, *Corynebacterium glutamicum* or *Basfia succiniproducens* can produce up to 105.8 g/l (89.5 mM) succinate mostly by sugar fermentation.^89^^,^^90^ *B. succiniproducens* can even produce succinate from waste glycerol.^91^^,^^92^ One of the highest succinate-yields form glucose (for wildtype organism) was reported for *A. succiniciproducens* with 0.97 succinate/glucose at optimal conditions.^90^^,^^93^ In this study, growing cells of *B. luti* achieved a yield of 0.42 succinate/glucose, which is similar to the succinate yield of *A. succiniciproducens* (0.41 succinate/glucose) at comparable pH.^93^ However, since the aim of this study was not to optimize succinate production, we do not know the true limit for succinate production by *B. luti*. By changing growth conditions such as pH or CO_2_ concentrations, higher succinate yields might be achieved.

In this study, we were able to elucidate novel features in the physiology of the genus *Blautia*, specifically for *Blautia* species without formate dehydrogenase. These bacteria seem to play in general a major role as a cornerstone in a healthy gut by the fermentation of carbohydrates and production of the short-chain fatty acid acetate, lactate, and succinate, but also by the production of formate and H_2_ in moderate amounts, which are central metabolites for cross-feeding in the gut. In addition, species of the genus *Blautia* might be able to prevent accumulation of toxic amounts of formate, CO or H_2_ in the gut. Therefore, it does not surprise that the genus *Blautia* is commonly associated with the human well-being.

## Supplementary Material

Trischler and Mueller Supplementary Data clean version.docx Trischler and Mueller Supplementary Data clean version.docx

## Data Availability

All data of this study are available within the article and its supplementary materials. The raw data is available from the authors upon reasonable request.

## References

[1] Turnbaugh PJ, Ley RE, Hamady M, Fraser-Liggett CM, Knight R, Gordon JI. The human microbiome project. Nature. 2007;449:804–810. doi: 10.1038/nature06244. 17943116 PMC3709439

[2] Ursell LK, Metcalf JL, Parfrey LW, Knight R. Defining the human microbiome. Nutr Rev. 2012;70:38–44. doi: 10.1111/j.1753-4887.2012.00493.x. PMC3426293 22861806

[3] Cresci GA, Bawden E. Gut microbiome: what we do and don't know. Nutr Clin Pract. 2015;30:734–746. doi: 10.1177/0884533615609899. 26449893 PMC4838018

[4] Hou K, Wu ZX, Chen XY, Wang JQ, Zhang D, Xiao C, Zhu D, Koya JB, Wei L, Li J. Microbiota in health and diseases. Signal Transduct Target Ther. 2022;7:135. doi: 10.1038/s41392-022-00974-4. 35461318 PMC9034083

[5] Parizadeh M, Arrieta MC. The global human gut microbiome: genes, lifestyles, and diet. Trends Mol Med. 2023;29:789–801. doi: 10.1016/j.molmed.2023.07.002. 37516570

[6] Van Hul M, Cani PD, Petitfils C, De Vos WM, Tilg H, El-Omar EM. What defines a healthy gut microbiome?. Gut. 2024;73:1893–1908. doi: 10.1136/gutjnl-2024-333378. 39322314 PMC11503168

[7] Holmberg SM, Feeney RH, Vishnu Prasoodanan PK, Puertolas-Balint F, Singh DK, Wongkuna S, Zandbergen L, Hauner H, Brandl B, Nieminen AI, Skurk T, et al. The gut commensal *Blautia* maintains colonic mucus function under low-fiber consumption through secretion of short-chain fatty acids. Nat Commun. 2024;15:3502. doi: 10.1038/s41467-024-47594-w. 38664378 PMC11045866

[8] Liu X, Mao B, Gu J, Wu J, Cui S, Wang G, Zhao J, Zhang H, Chen W. *Blautia*-a new functional genus with potential probiotic properties?. Gut Microbes. 2021;13:1–21. doi: 10.1080/19490976.2021.1875796. PMC7872077 33525961

[9] Drake HL, Gößner AS, Daniel SL. Old acetogens, new light. Ann N Y Acad Sci. 2008;1125:100–128. doi: 10.1196/annals.1419.016. 18378590

[10] Müller V. Energy conservation in acetogenic bacteria. Appl Environ Microbiol. 2003;69:6345–6353. doi: 10.1128/aem.69.11.6345-6353.2003. 14602585 PMC262307

[11] Müller V, Inkamp F, Rauwolf A, Küsel K, Drake HL. Molecular and cellular biology of acetogenic bacteria. In M Nakano, P Zuber (Eds.), Strict and facultative anaerobes: medical and environmental aspects. 2004. p. 251–281 Norfolk: Horizon Scientific Press.

[12] Ragsdale SW. Enzymology of the Wood-Ljungdahl pathway of acetogenesis. Ann NY Acad Sci. 2008;1125:129–136. doi: 10.1196/annals.1419.015. 18378591 PMC3040112

[13] Pezacka E, Wood HG. Role of carbon monoxide dehydrogenase in the autotrophic pathway used by acetogenic bacteria. Proc Natl Acad Sci USA. 1984a;81:6261–6265. doi: 10.1073/pnas.81.20.6261. 6436811 PMC391903

[14] Pezacka E, Wood HG. The synthesis of acetyl-CoA by *Clostridium thermoaceticum* from carbon dioxide, hydrogen, coenzyme A and methyltetrahydrofolate. Arch Microbiol. 1984b;137:63–69. doi: 10.1007/BF00425809. 6424623

[15] Seravalli J, Kumar M, Lu WP, Ragsdale SW. Mechanism of carbon monoxide oxidation by the carbon monoxide dehydrogenase/acetyl-CoA synthase from *Clostridium thermoaceticum*: kinetic characterization of the intermediates. Biochemistry. 1997;36:11241–11251. doi: 10.1021/bi970590m. 9287167

[16] Schuchmann K, Müller V. Autotrophy at the thermodynamic limit of life: a model for energy conservation in acetogenic bacteria. Nat Rev Microbiol. 2014;12:809–821. doi: 10.1038/nrmicro3365. 25383604

[17] Fischer F, Lieske R, Winzler K. Biologische Gasreaktionen. II. Über die Bildung von Essigsäure bei der biologischen Umsetzung von Kohlenoxyd und Kohlensäure zu Methan. Biochem Z. 1932;245:2–12.

[18] Schaupp A, Ljungdahl LG. Purification and properties of acetate kinase from *Clostridium thermoaceticum*. Arch Microbiol. 1974;100:121–129. doi: 10.1007/BF00446312. 4447427

[19] Thauer RK, Jungermann K, Decker K. Energy conservation in chemotrophic anaerobic bacteria. Bacteriol Rev. 1977;41:100–180. doi: 10.1128/br.41.1.100-180.1977. 860983 PMC413997

[20] Fuchs G. Alternative pathways of carbon dioxide fixation: insights into the early evolution of life?. Annu Rev Microbiol. 2011;65:631–658. doi: 10.1146/annurev-micro-090110-102801. 21740227

[21] Buckel W, Thauer RK. Energy conservation *via* electron bifurcating ferredoxin reduction and proton/Na^+^ translocating ferredoxin oxidation. Biochim Biophys Acta. 2013;1827:94–113. doi: 10.1016/j.bbabio.2012.07.002. 22800682

[22] Müller V, Chowdhury NP, Basen M. Electron bifurcation: a long-hidden energy-coupling mechanism. Annu Rev Microbiol. 2018;72:331–353. doi: 10.1146/annurev-micro-090816-093440. 29924687

[23] Katsyv A, Kumar A, Saura P, Poverlein MC, Freibert SA, Stripp ST, Jain S, Gamiz-Hernandez AP, Kaila VRI, Müller V, et al. Molecular basis of the electron bifurcation mechanism in the [FeFe]-hydrogenase complex HydABC. J Am Chem Soc. 2023;145:5696–5709. doi: 10.1021/jacs.2c11683. 36811855 PMC10021017

[24] Schuchmann K, Chowdhury NP, Müller V. Complex multimeric [FeFe] hydrogenases: biochemistry, physiology and new opportunities for the hydrogen economy. Front microbiol. 2018;9:2911. doi: 10.3389/fmicb.2018.02911. 30564206 PMC6288185

[25] Kremp F, Roth J, Müller V. The *Sporomusa* type Nfn is a novel type of electron-bifurcating transhydrogenase that links the redox pools in acetogenic bacteria. Sci Rep. 2020;10:14872. doi: 10.1038/s41598-020-71038-2. 32913242 PMC7483475

[26] Wang S, Huang H, Moll J, Thauer RK. NADP^+^ reduction with reduced ferredoxin and NADP^+^ reduction with NADH are coupled *via* an electron bifurcating enzyme complex in *Clostridium kluyveri*. J Bacteriol. 2010;192:5115–5123. doi: 10.1128/JB.00612-10. 20675474 PMC2944534

[27] Bache R, Pfennig N. Selective isolation of *Acetobacterium woodii* on methoxylated aromatic acids and determination of growth yields. Arch Microbiol. 1981;130:255–261. doi: 10.1007/BF00459530.

[28] Buschhorn H, Dürre P, Gottschalk G. Production and utilization of ethanol by the homoacetogen *Acetobacterium woodii*. Appl Environ Microbiol. 1989;55:1835–1840. doi: 10.1128/aem.55.7.1835-1840.1989. 16347978 PMC202959

[29] Dönig J, Müller V. Alanine, a novel growth substrate for the acetogenic bacterium *Acetobacterium woodii*. Appl Environ Microbiol. 2018;84:e02023–02018. doi: 10.1128/AEM.02023-18. PMC6238063 30242008

[30] Drake HL, Daniel S, Küsel K, Matthies C, Kuhner C, Braus-Strohmeyer S. Acetogenic bacteria: what are the *in situ* consequences of their diverse metabolic diversities? Biofactors. 1997;6(1):13–24. doi: 10.1002/biof.5520060103. 9233536

[31] Eichler B, Schink B. Oxidation of primary aliphatic alcohols by *Acetobacterium carbinolicum* sp. nov., a homoacetogenic anaerobe. Arch Microbiol. 1984;140:147–152. doi: 10.1007/BF00454917.

[32] Hess V, Oyrik O, Trifunovic D, Müller V. 2,3-butanediol metabolism in the acetogen *Acetobacterium woodii*. Appl Environ Microbiol. 2015;81:4711–4719. doi: 10.1128/AEM.00960-15. 25934628 PMC4551200

[33] Lechtenfeld M, Heine J, Sameith J, Kremp F, Müller V. Glycine betaine metabolism in the acetogenic bacterium *Acetobacterium woodii*. Environ Microbiol. 2018;20:4512–4525. doi: 10.1111/1462-2920.14389. 30136352

[34] Moon J, Henke L, Merz N, Basen M. A thermostable mannitol-1-phosphate dehydrogenase is required in mannitol metabolism of the thermophilic acetogenic bacterium *Thermoanaerobacter kivui*. Environ Microbiol. 2019;21:3728–3736. doi: 10.1111/1462-2920.14720. 31219674

[35] Schuchmann K, Schmidt S, Martinez Lopez A, Kaberline C, Kuhns M, Lorenzen W, Bode HB, Joos F, Müller V, Metcalf WW. Nonacetogenic growth of the acetogen *Acetobacterium woodii* on 1,2-propanediol. J Bacteriol. 2015;197:382–391. doi: 10.1128/JB.02383-14. 25384483 PMC4272584

[36] Seifritz C, Fröstl JM, Drake HL, Daniel SL. Glycolate as a metabolic substrate for the acetogen *Moorella thermoacetica*. FEMS Microbiol Lett. 1999;170:399–405. doi: 10.1111/j.1574-6968.1999.tb13400.x.

[37] Trifunović D, Schuchmann K, Müller V. Ethylene glycol metabolism in the acetogen *Acetobacterium woodii*. J Bacteriol. 2016;198:1058–1065. doi: 10.1128/JB.00942-15. 26787767 PMC4800866

[38] Trischler R, Roth J, Sorbara MT, Schlegel X, Müller V. A functional Wood-Ljungdahl pathway devoid of a formate dehydrogenase in the gut acetogens *Blautia wexlerae*, *Blautia luti* and beyond. Environ Microbiol. 2022;24:3111–3123. doi: 10.1111/1462-2920.16029. 35466558

[39] Heise R, Müller V, Gottschalk G. Sodium dependence of acetate formation by the acetogenic bacterium *Acetobacterium woodii*. J Bacteriol. 1989;171:5473–5478. doi: 10.1128/jb.171.10.5473-5478.1989. 2507527 PMC210386

[40] Trischler R, Poehlein A, Daniel R, Müller V. Ethanologenesis from glycerol by the gut acetogen *Blautia schinkii*. Environ Microbiol. 2023;25:3577–3591. doi: 10.1111/1462-2920.16517. 37807918

[41] Schmidt K, Liaaen-Jensen S, Schlegel HG. Die Carotinoide der *Thiorhodaceae*. Arch Microbiol. 1963;46:117–126. doi: 10.1007/BF00408204. 14044829

[42] Weghoff MC, Müller V. CO metabolism in the thermophilic acetogen *Thermoanaerobacter kivui*. Appl Environ Microbiol. 2016;82:2312–2319. doi: 10.1128/AEM.00122-16. 26850300 PMC4959504

[43] Bertsch J, Müller V. CO metabolism in the acetogen *Acetobacterium woodii*. Appl Environ Microbiol. 2015;81:5949–5956. doi: 10.1128/AEM.01772-15. 26092462 PMC4551271

[44] Bradford MM. A rapid and sensitive method for the quantification of microgram quantities of protein utilizing the principle of proteine-dye-binding. Anal Biochem. 1976;72:248–254. doi: 10.1006/abio.1976.9999. 942051

[45] Bertsch J, Siemund AL, Kremp F, Müller V. A novel route for ethanol oxidation in the acetogenic bacterium *Acetobacterium woodii*: The acetaldehyde/ethanol dehydrogenase pathway. Environ Microbiol. 2016;18:2913–2922. doi: 10.1111/1462-2920.13082. 26472176

[46] Simmering R, Taras D, Schwiertz A, Le Blay G, Gruhl B, Lawson PA, Collins MD, Blaut M. *Ruminococcus luti* sp. nov., isolated from a human faecal sample. Syst Appl Microbiol. 2002;25:189–193. doi: 10.1078/0723-2020-00112. 12353871

[47] Dostal A, Lacroix C, Bircher L, Pham VT, Follador R, Zimmermann MB, Chassard C. Iron modulates butyrate production by a child gut microbiota *in vitro*. mBio. 2015;6:e01453–01415. doi: 10.1128/mBio.01453-15. 26578675 PMC4659462

[48] Bittrich S, Segura J, Duarte JM, Burley SK, Rose Y. RCSB protein data bank: exploring protein 3D similarities *via* comprehensive structural alignments. Bioinformatics. 2024;40:btae370. doi: 10.1093/bioinformatics/btae370. 38870521 PMC11212067

[49] Welsh C, Cabotaje PR, Marcelino VR, Watts TD, Kountz DJ, Gould JA, et al. A widespread hydrogenase drives fermentative growth of gut bacteria in healthy people. bioRxiv. 2024. 608110. doi: 10.1101/2024.08.15.608110. PMC12578642 41131367

[50] Touyama M, Jin JS, Kibe R, Hayashi H, Benno Y. Quantification of *Blautia wexlerae* and *Blautia luti* in human faeces by real-time PCR using specific primers. Benef Microbes. 2015;6:583–590. doi: 10.3920/BM2014.0133. 25691104

[51] Ljungdahl LG. The autotrophic pathway of acetate synthesis in acetogenic bacteria. Ann Rev Microbiol. 1986;40:415–450. doi: 10.1146/annurev.mi.40.100186.002215. 3096193

[52] Wood HG, Ragsdale SW, Pezacka E. The acetyl-CoA pathway of autotrophic growth. FEMS Microbiol Rev. 1986;39:345–362. doi: 10.1016/0378-1097(86)90022-4.

[53] Moon J, Donig J, Kramer S, Poehlein A, Daniel R, Müller V. Formate metabolism in the acetogenic bacterium *Acetobacterium woodii*. Environ Microbiol. 2021;23:4214–4227. doi: 10.1111/1462-2920.15598. 33989450

[54] Müller V. New horizons in acetogenic conversion of one-carbon substrates and biological hydrogen storage. Trends Biotechnol. 2019;37:1344–1354. doi: 10.1016/j.tibtech.2019.05.008. 31257058

[55] Schuchmann K, Müller V. Energetics and application of heterotrophy in acetogenic bacteria. Appl Environ Microbiol. 2016;82:4056–4069. doi: 10.1128/AEM.00882-16. 27208103 PMC4959221

[56] Wiechmann A, Ciurus S, Oswald F, Seiler VN, Müller V. It does not always take two to tango: “Syntrophy”​​​​​​ *via* hydrogen cycling in one bacterial cell. ISME J. 2020;14:1561–1570. doi: 10.1038/s41396-020-0627-1. 32203116 PMC7242416

[57] Smith NW, Shorten PR, Altermann EH, Roy NC, McNabb WC. Hydrogen cross-feeders of the human gastrointestinal tract. Gut Microbes. 2019;10:270–288. doi: 10.1080/19490976.2018.1546522. 30563420 PMC6546324

[58] Hylemon PB, Harris SC, Ridlon JM. Metabolism of hydrogen gases and bile acids in the gut microbiome. FEBS Lett. 2018;592:2070–2082. doi: 10.1002/1873-3468.13064. 29683480

[59] Yao Y, Fu B, Han D, Zhang Y, Liu H. Formate-dependent acetogenic utilization of glucose by the fecal acetogen *Clostridium bovifaecis*. Appl Environ Microbiol. 2020;86:e01870–01820. doi: 10.1128/AEM.01870-20. 32948524 PMC7657615

[60] Jain S, Dietrich HM, Müller V, Basen M. Formate is required for growth of the thermophilic acetogenic bacterium *Thermoanaerobacter kivui* lacking hydrogen-dependent carbon dioxide reductase (HDCR). Front Microbiol. 2020;11:59. doi: 10.3389/fmicb.2020.00059. 32082286 PMC7005907

[61] Moon J, Schubert A, Poehlein A, Daniel R, Müller V. A new metabolic trait in an acetogen: mixed acid fermentation of fructose in a methylene-tetrahydrofolate reductase mutant of *Acetobacterium woodii*. Environ Microbiol Rep. 2023b;15:339–351. doi: 10.1111/1758-2229.13160. 37150590 PMC10472528

[62] Moon J, Waschinger LM, Müller V. Lactate formation from fructose or C1 compounds in the acetogen *Acetobacterium woodii* by metabolic engineering. Appl Microbiol Biotechnol. 2023a;107:5491–5502. doi: 10.1007/s00253-023-12637-7. 37417977 PMC10390620

[63] Metz G, Jenkins DJ, Peters TJ, Newman A, Blendis LM. Breath hydrogen as a diagnostic method for hypolactasia. Lancet. 1975;1:1155–1157. doi: 10.1016/s0140-6736(75)93135-9. 48774

[64] Rhodes JM, Middleton P, Jewell DP. The lactulose hydrogen breath test as a diagnostic test for small-bowel bacterial overgrowth. Scand J Gastroenterol. 1979;14:333–336. doi: 10.3109/00365527909179892. 441681

[65] Simren M, Stotzer PO. Use and abuse of hydrogen breath tests. Gut. 2006;55:297–303. doi: 10.1136/gut.2005.075127. 16474100 PMC1856094

[66] Ge L, Yang M, Yang NN, Yin XX, Song WG. Molecular hydrogen: a preventive and therapeutic medical gas for various diseases. Oncotarget. 2017;8:102653–102673. doi: 10.18632/oncotarget.21130. 29254278 PMC5731988

[67] Korovljev D, Trivic T, Drid P, Ostojic SM. Molecular hydrogen affects body composition, metabolic profiles, and mitochondrial function in middle-aged overweight women. Ir J Med Sci. 2018;187:85–89. doi: 10.1007/s11845-017-1638-4. 28560519

[68] Ohta S. Molecular hydrogen as a preventive and therapeutic medical gas: initiation, development and potential of hydrogen medicine. Pharmacol Ther. 2014;144:1–11. doi: 10.1016/j.pharmthera.2014.04.006. 24769081

[69] Fonty G, Joblin K, Chavarot M, Roux R, Naylor G, Michallon F. Establishment and development of ruminal hydrogenotrophs in methanogen-free lambs. Appl Environ Microbiol. 2007;73:6391–6403. doi: 10.1128/AEM.00181-07. 17675444 PMC2075041

[70] Hopper CP, De La Cruz LK, Lyles KV, Wareham LK, Gilbert JA, Eichenbaum Z, Magierowski M, Poole RK, Wollborn J, Wang B. Role of carbon monoxide in host-gut microbiome communication. Chem Rev. 2020;120:13273–13311. doi: 10.1021/acs.chemrev.0c00586. 33089988

[71] Morse D, Choi AM. Heme oxygenase-1: from bench to bedside. Am J Respir Crit Care Med. 2005;172:660–670. doi: 10.1164/rccm.200404-465SO. 15901614

[72] Otterbein LE, Bach FH, Alam J, Soares M, Tao Lu H, Wysk M, Davis RJ, Flavell RA, Choi AM. Carbon monoxide has anti-inflammatory effects involving the mitogen-activated protein kinase pathway. Nat Med. 2000;6:422–428. doi: 10.1038/74680. 10742149

[73] Maines MD. Carbon monoxide: an emerging regulator of cGMP in the brain. Mol Cell Neurosci. 1993;4:389–397. doi: 10.1006/mcne.1993.1049. 19912945

[74] Snyder SH, Jaffrey SR, Zakhary R. Nitric oxide and carbon monoxide: parallel roles as neural messengers. Brain Res Brain Res Rev. 1998;26:167–175. doi: 10.1016/s0165-0173(97)00032-5. 9651518

[75] Onyiah JC, Sheikh SZ, Maharshak N, Otterbein LE, Plevy SE. Heme oxygenase-1 and carbon monoxide regulate intestinal homeostasis and mucosal immune responses to the enteric microbiota. Gut Microbes. 2014;5:220–224. doi: 10.4161/gmic.27290. 24637794 PMC4063848

[76] Ernst A, Zibrak JD. Carbon monoxide poisoning. N Engl J Med. 1998;339:1603–1608. doi: 10.1056/NEJM199811263392206. 9828249

[77] Townsend CL, Maynard RL. Effects on health of prolonged exposure to low concentrations of carbon monoxide. Occup Environ Med. 2002;59:708–711. doi: 10.1136/oem.59.10.708. 12356933 PMC1740215

[78] Roy R, Andaluri G, Miller W. Carbon monoxide signature in human body: measured data and gis physiology. J Eng Technol Res. 2016;5:5–7.

[79] Katayama YA, Kamikawa R, Yoshida T. Phylogenetic diversity of putative nickel-containing carbon monoxide dehydrogenase-encoding prokaryotes in the human gut microbiome. Microb Genom. 2024;10:001285. doi: 10.1099/mgen.0.001285. 39166974 PMC11338639

[80] Wei YH, Ma X, Zhao JC, Wang XQ, Gao CQ. Succinate metabolism and its regulation of host-microbe interactions. Gut Microbes. 2023;15:2190300. doi: 10.1080/19490976.2023.2190300. 36946592 PMC10038034

[81] Ives SJ, Zaleski KS, Slocum C, Escudero D, Sheridan C, Legesse S, Vidal K, Lagalwar S, Reynolds TH. The effect of succinic acid on the metabolic profile in high-fat diet-induced obesity and insulin resistance. Physiol Rep. 2020;8:e14630. doi: 10.14814/phy2.14630. 33185326 PMC7663994

[82] Fischbach MA, Sonnenburg JL. Eating for two: how metabolism establishes interspecies interactions in the gut. Cell Host Microbe. 2011;10:336–347. doi: 10.1016/j.chom.2011.10.002. 22018234 PMC3225337

[83] Cummings JH, Pomare EW, Branch WJ, Naylor CP, Macfarlane GT. Short chain fatty acids in human large intestine, portal, hepatic and venous blood. Gut. 1987;28:1221–1227. doi: 10.1136/gut.28.10.1221. 3678950 PMC1433442

[84] Meijer-Severs GJ, van Santen E. Short-chain fatty acids and succinate in feces of healthy human volunteers and their correlation with anaerobe cultural counts. Scand J Gastroenterol. 1987;22:672–676. doi: 10.3109/00365528709011141. 3659829

[85] Osaka T, Moriyama E, Arai S, Date Y, Yagi J, Kikuchi J, Tsuneda S. Meta-analysis of fecal microbiota and metabolites in experimental colitic mice during the inflammatory and healing phases. Nutrients. 2017;9:1329. doi: 10.3390/nu9121329. 29211010 PMC5748779

[86] Macias-Ceja DC, Ortiz-Masia D, Salvador P, Gisbert-Ferrandiz L, Hernandez C, Hausmann M, Ortiz-Masiá D, Gisbert-Ferrándiz L, Hernández C, Rogler G, et al. Succinate receptor mediates intestinal inflammation and fibrosis. Mucosal Immunol. 2019;12:178–187. doi: 10.1038/s41385-018-0087-3. 30279517

[87] Mills E, O'Neill LA. Succinate: a metabolic signal in inflammation. Trends Cell Biol. 2014;24:313–320. doi: 10.1016/j.tcb.2013.11.008. 24361092

[88] Werpy TA, Holladay JE, White JF. Top value added chemicals from biomass: I. Results of screening for potential candidates from sugars and synthesis gas. 2004. Springfield (VA): U.S. Department of Energy. 68. p. Report No.: PNNL-14808.

[89] Mitrea L, Teleky BE, Nemes SA, Plamada D, Varvara RA, Pascuta MS, Ciont C, Cocean A, Medeleanu M, Nistor A, et al. Succinic acid - a run-through of the latest perspectives of production from renewable biomass. Heliyon. 2024;10:e25551. doi: 10.1016/j.heliyon.2024.e25551. 38327454 PMC10848017

[90] Song H, Lee SY. Production of succinic acid by bacterial fermentation. Enzyme Microb Technol. 2006;39:352–361. doi: 10.1016/j.enzmictec.2005.11.043.

[91] Scholten E, Dagele D. Succinic acid production by a newly isolated bacterium. Biotechnol Lett. 2008;30:2143–2146. doi: 10.1007/s10529-008-9806-2. 18651227

[92] Scholten E, Renz T, Thomas J. Continuous cultivation approach for fermentative succinic acid production from crude glycerol by *Basfia succiniciproducens* DD1. Biotechnol Lett. 2009;31:1947–1951. doi: 10.1007/s10529-009-0104-4. 19705071

[93] Nghiem NP, Davison BH, Suttle BE, Richardson GR. Production of succinic acid by *Anaerobiospirillum succiniciproducens*. Appl Biochem Biotechnol. 1997;63–65:565–576. doi: 10.1007/BF02920454. 18576111

