# Supplementary material for: Formate as electron carrier in the gut acetogen Blautia luti: a model for electron transfer in the gut microbiome
Source: Gut Microbes. 2026 Jan 2;18(1):2609406. doi: 10.1080/19490976.2025.2609406 (PMC12773636; doi:10.1080/19490976.2025.2609406)
Supplement: Trischler and Mueller Supplementary Data clean version.docx [file KGMI_A_2609406_SM7348.docx]

**Supplementary data**

**Formate as electron carrier in the gut acetogen *Blautia luti*: a model for electron transfer in the gut microbiome**

Raphael Trischler^1^ and Volker Müller^1#^

^1^ *Molecular Microbiology & Bioenergetics, Institute of Molecular Biosciences, Johann Wolfgang Goethe University, Max-von-Laue Str. 9, D-60438 Frankfurt, Germany*

*#Corresponding author Mailing address: Department of Molecular Microbiology & Bioenergetics, Institute of Molecular Biosciences, Johann Wolfgang Goethe University, Max-von-Laue-Str 9, D-60438 Frankfurt, Germany Phone: 49-69-79829507 Fax: 49-69-79829306 E-mail:* [*vmueller@biouni-frankfurt.de*](mailto:vmueller@biouni-frankfurt.de)


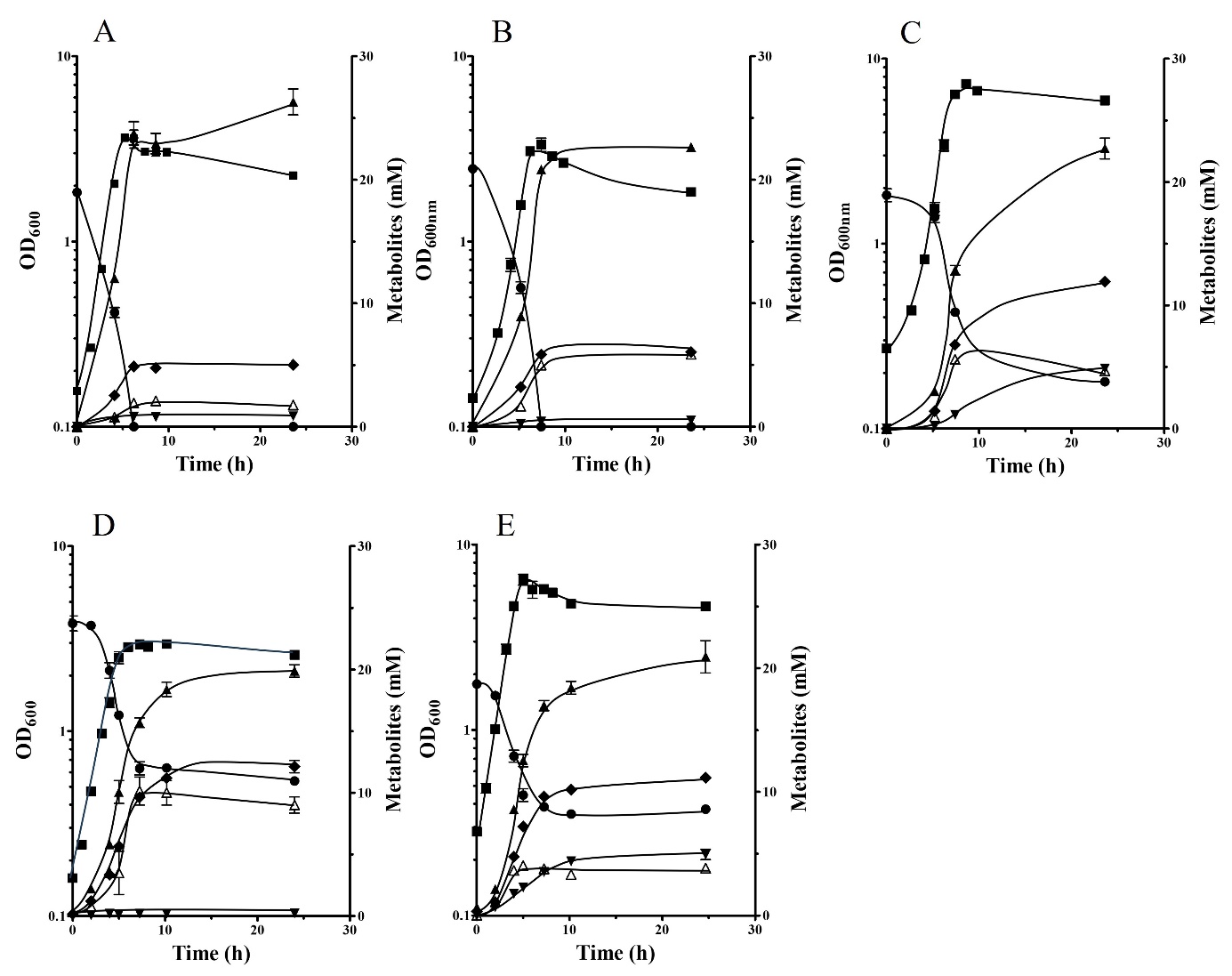


**Supplementary Fig. S1. Growth of *B. luti* with different carbohydrates as carbon and energy sources.** *B. luti* was grown in serum flasks containing 50 ml CO_2_/KHCO_3_-buffered complex medium at 37°C with 20 mM of xylose (A), arabinose (B), sucrose (C), trehalose (D) and raffinose (E). The pre-culture used to inoculate (5 %) the medium was transferred three times to each growth substrate. The optical densities at 600 nm (■) and the concentrations of acetate (▲), succinate (♦), lactate (▼), formate (Δ) and the corresponding carbohydrate (●) were determined. All data points mean ± SEM; *N* = 3 independent experiments.


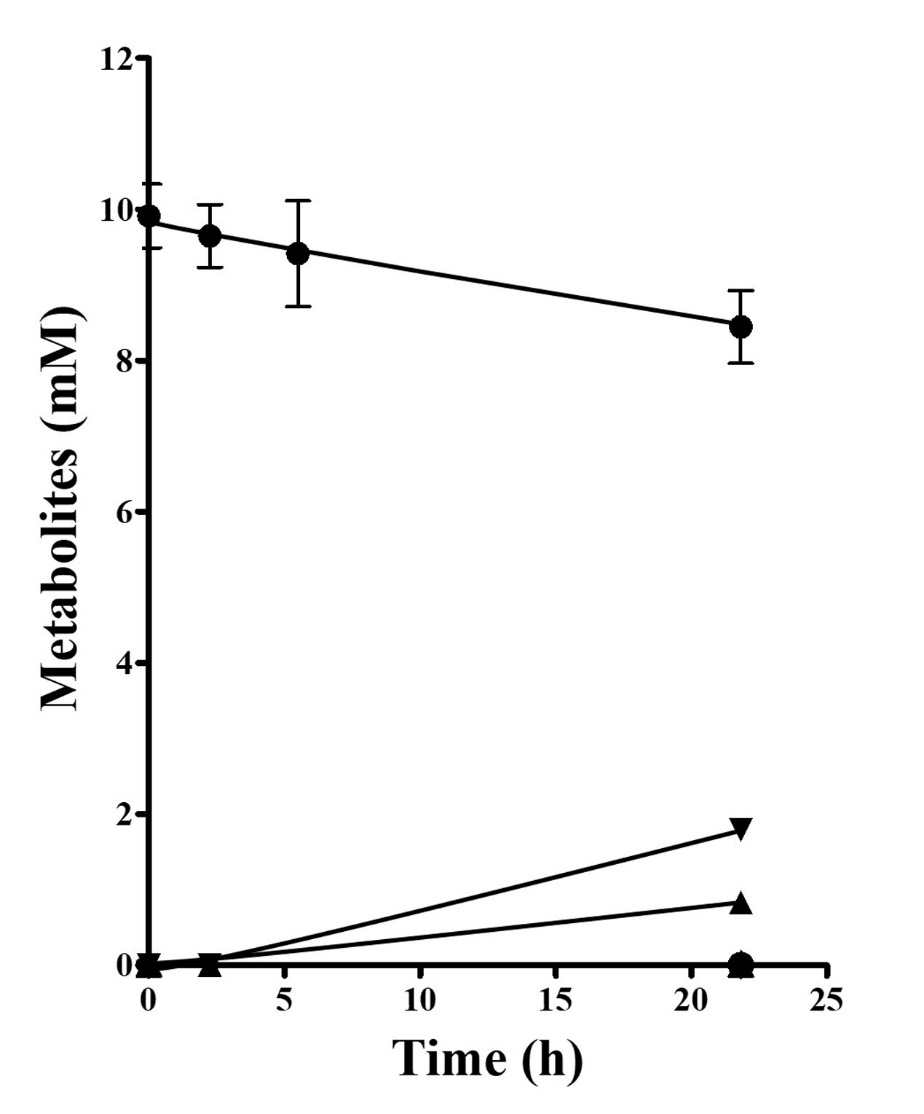


**Supplementary Fig. S2. Fermentation of glucose by resting cells of *B. luti* in the absence of CO_2_/KHCO_3_.** *B. luti* was grown in CO_2_/KHCO_3_-buffered medium with 20 mM glucose as substrate at 37°C to late exponential growth phase, harvested, washed and resuspended in 10 ml imidazole buffer (50 mM imidazole, 20 mM NaCl, 20 mM MgSO_4_, 2 mM DET, 4.4 µM resazurin, pH 7.0) to a final protein concentration of 1 mg ml^-1^ in 115 ml serum flasks under an atmosphere of 100 % N_2_. The concentrations of glucose (●), acetate (▲), succinate (♦), formate (Δ), lactate (▼) and H_2_ (○) were determined. Each data point are mean ± SEM; *N* = 2 independent experiments.


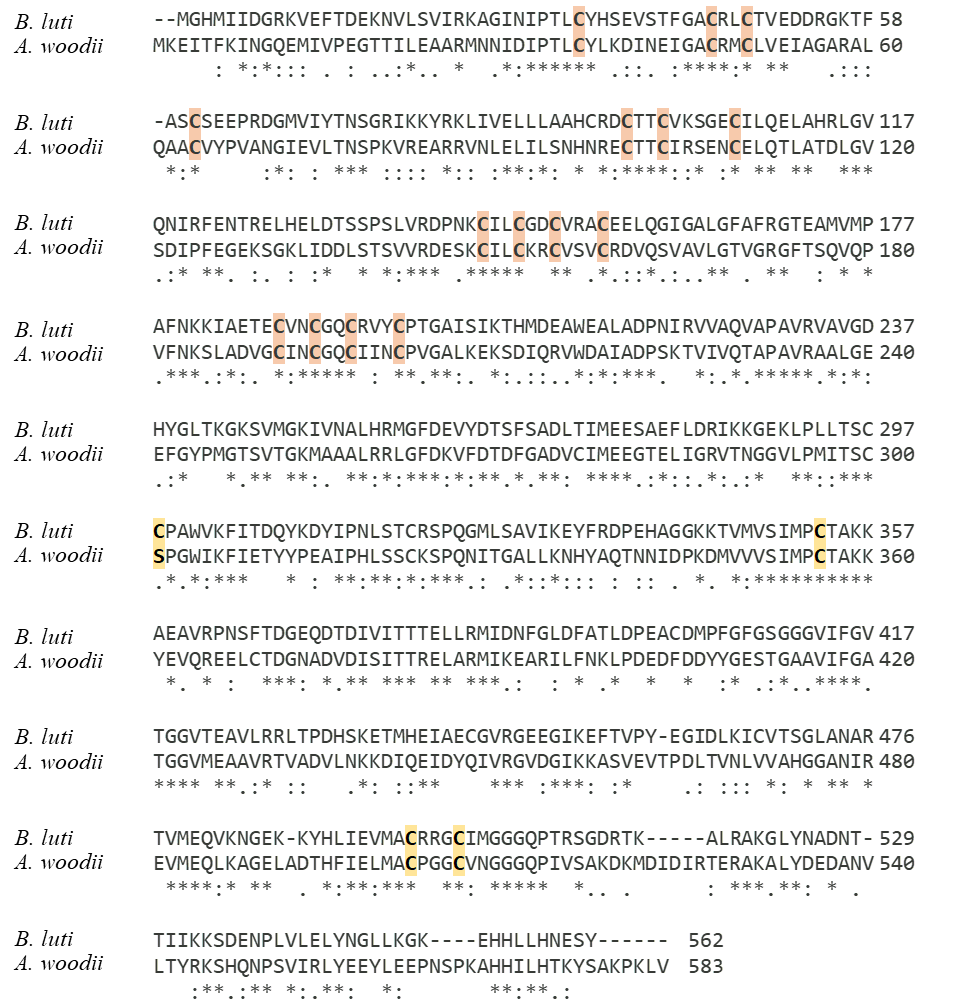


**Supplementary Fig. S3. Sequence alignment of the electron-bifurcating hydrogenase subunit HydA of *B. luti* and *A. woodii*.** Amino acids responsible for FeS-cluster (orange) and H-cluster (yellow) coordination are highlighted. Conserved amino acids are indicated by asterisk. The sequence alignment was performed with ClustalOmega.


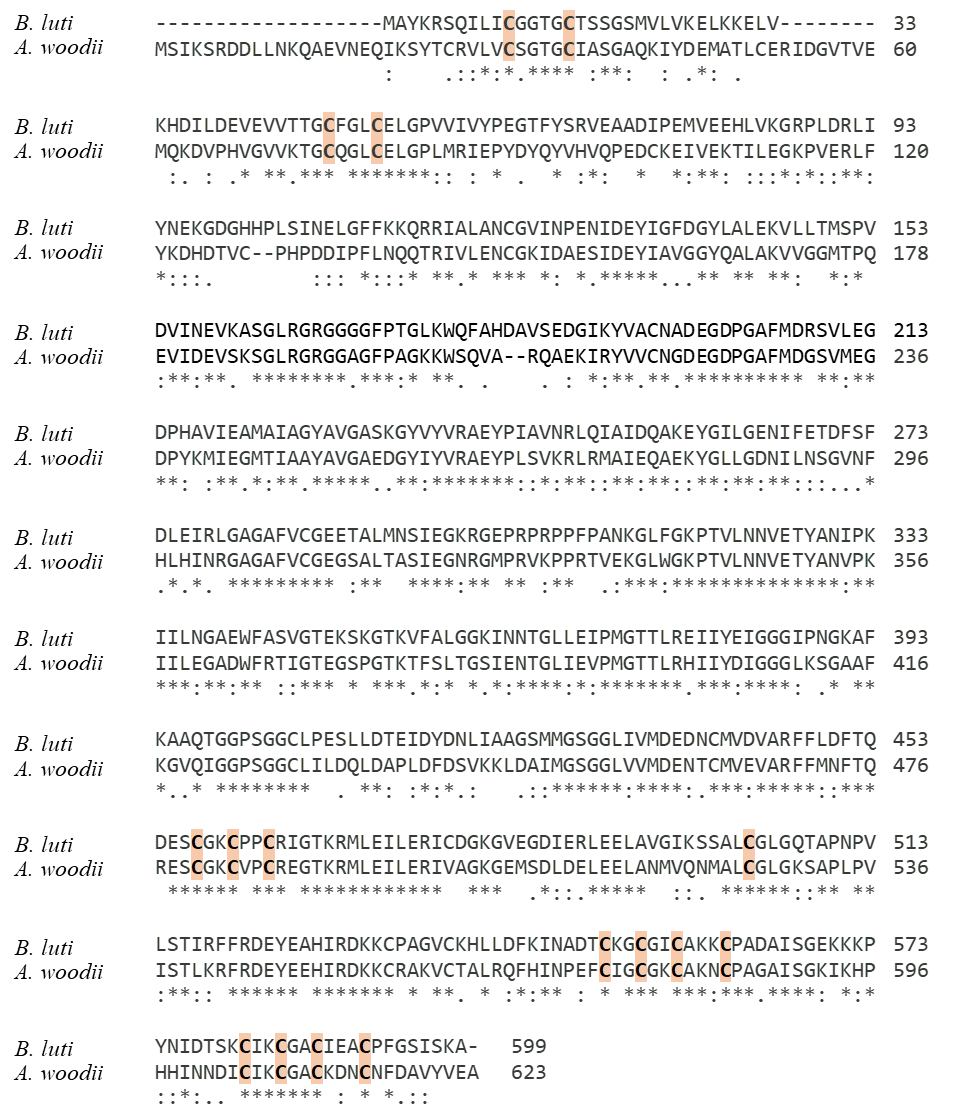


**Supplementary Fig. S4. Sequence alignment of the electron-bifurcating hydrogenase subunit HydB of *B. luti* and *A. woodii*.** Amino acids responsible for FeS-cluster coordination are highlighted. Conserved amino acids are indicated by asterisk. The sequence alignment was performed with ClustalOmega.


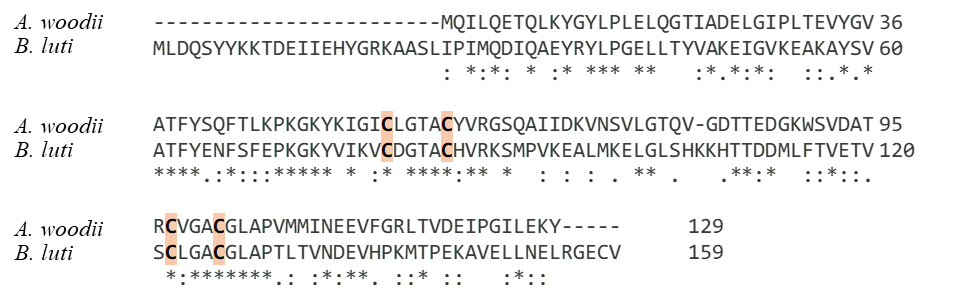


**Supplementary Fig. S5. Sequence alignment of the electron-bifurcating hydrogenase subunit HydC of *B. luti* and *A. woodii*.** Amino acids responsible for FeS-cluster coordination are highlighted. Conserved amino acids are indicated by asterisk. The sequence alignment was performed with ClustalOmega.


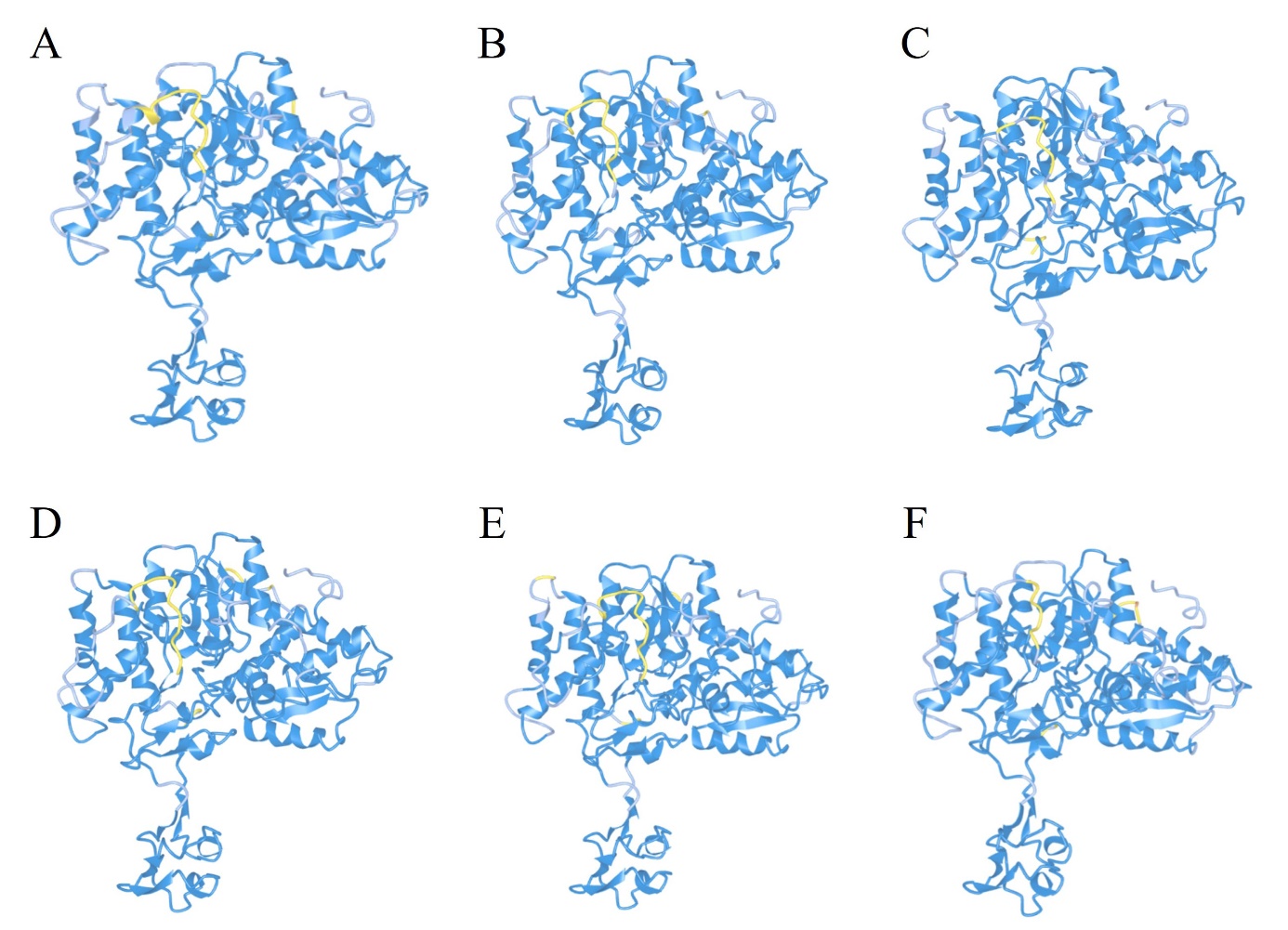


**Supplementary Fig. S6. Comparison of predicted structures of HydM-type hydrogenases.** Predicted structures of HydM of *Blautia obeum* (A), *Blautia hydrogenotrophica* (B), *Blautia coccoides* (C), *Blautia caecimuris* (D), *Blautia producta* (E) and *Blautia massiliensis* (F). Structure preductions were performed by RCSB protein Data Bank (Bittrich et al., 2024).

**
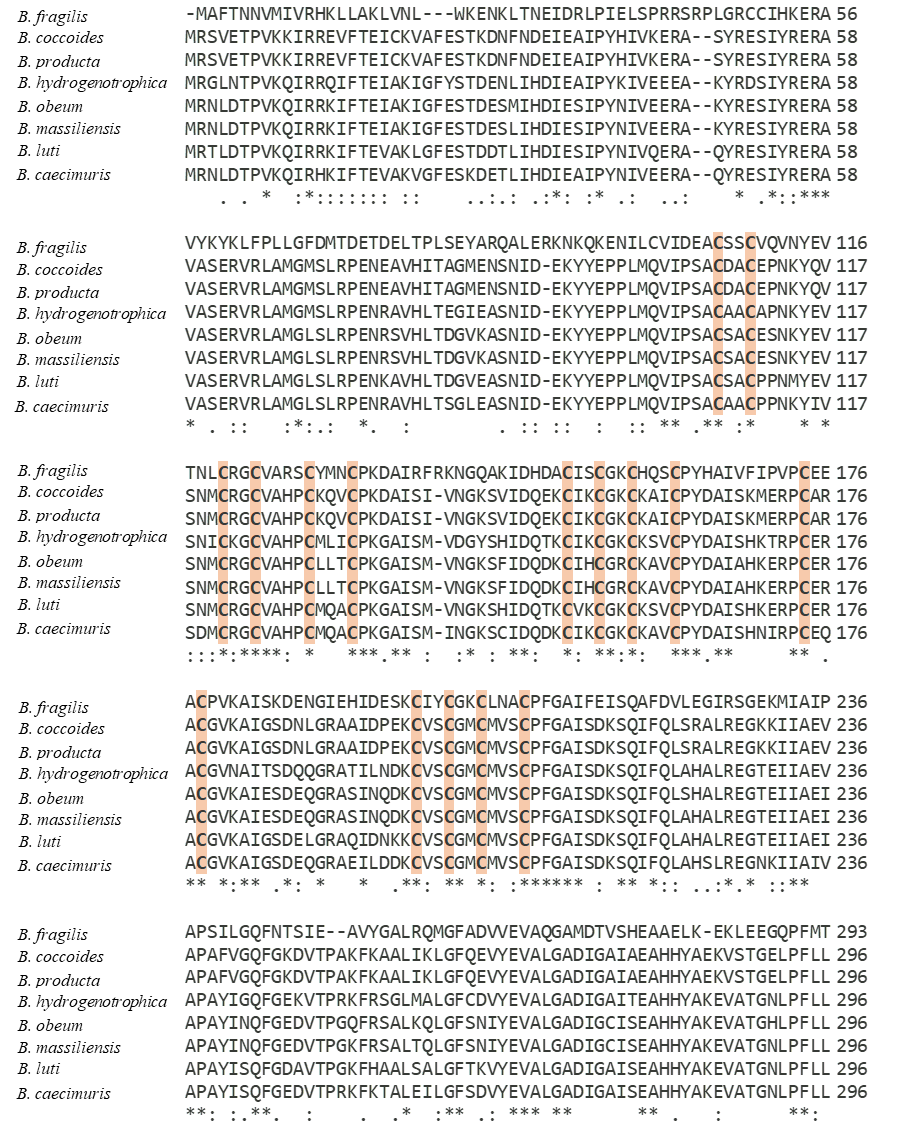
**

**
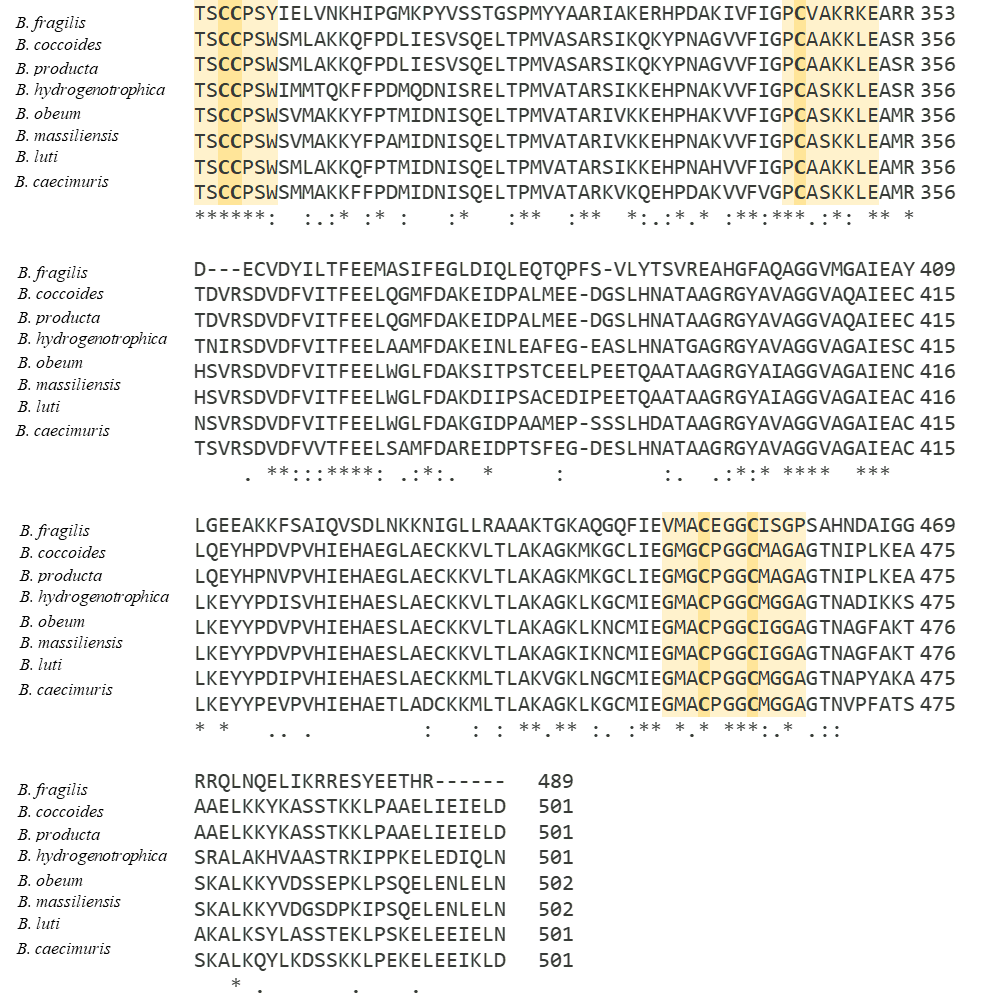
**

**Supplementary Fig. S7. Sequence alignment of HydM of different *Blautia* species and *B. fragilis*.** Amino acids responsible for FeS-cluster (orange) and H-cluster (yellow) coordination are highlighted. Conserved amino acids are indicated by asterisk. The sequence alignment was performed with ClustalOmega.


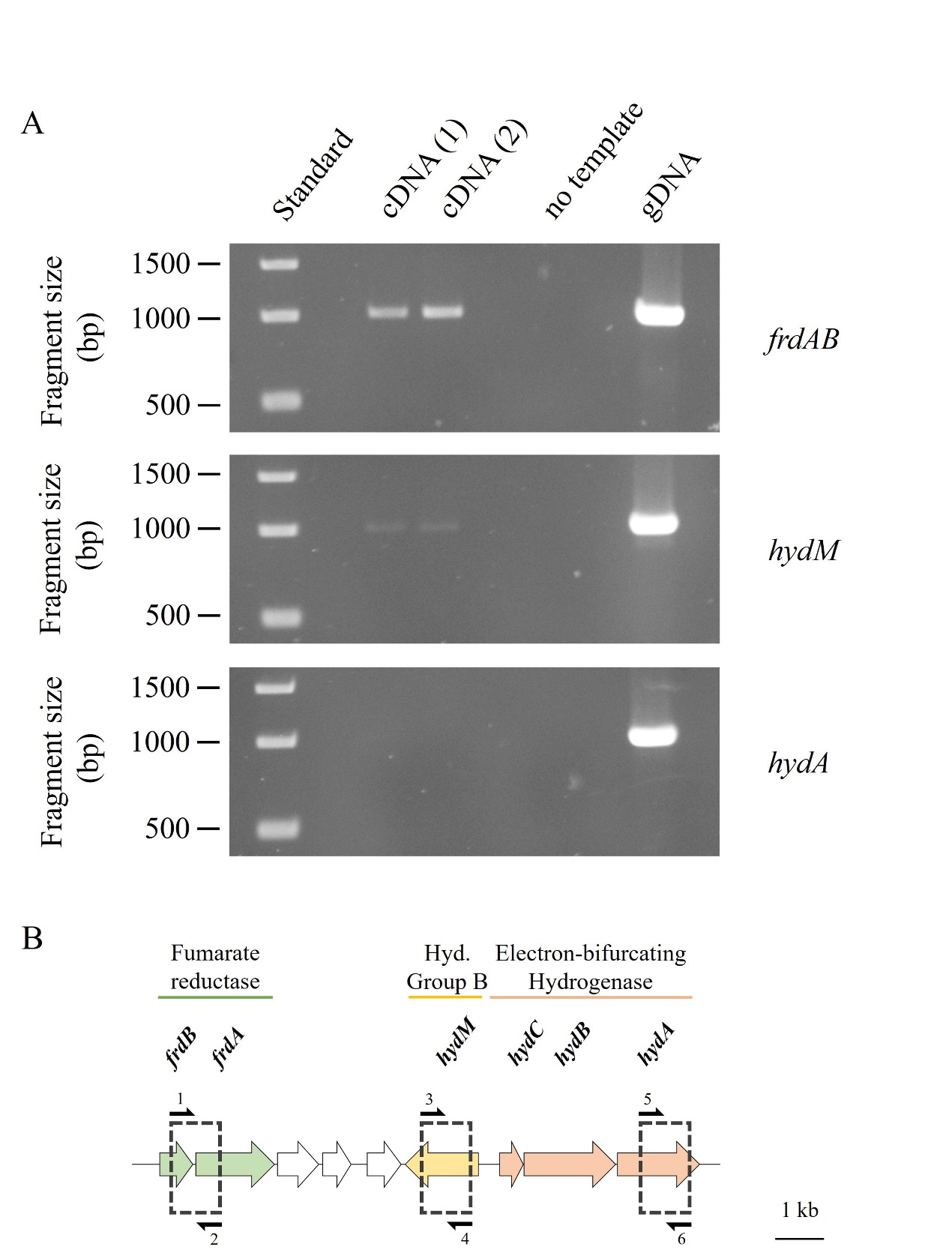


**Supplementary Fig. S8. Transcript abundance of hydrogenase encoding genes in *B. luti* during glucose fermentation.** (A) *B. luti* was grown in CO_2_/KHCO_3_-buffered complex media with 20 mM glucose as substrate to mid exponential growth phase. Cells were harvested and mRNA was isolated for cDNA synthesis. The transcript abundance of the hydrogenase encoding genes *hydM* and *hydA* as well as the fumarate reductase encoding genes *frdAB* was analysed with semi-quantitative PCR with two independent cDNAs as template. Controls contained no template or genomic DNA (gDNA). The data shown is representative for two independent experiments. (B) Binding sites for primer used for semi-quantitative PCR in the genome of *B. luti*. The primer used in this study are listed in supplementary Tab. S1.

**Supplementary Tab. S1. Primer used in this study.**

| **Primer** | **Sequence (5´→ 3´)** |
| --- | --- |
| 1 | GAAGACCTGAAACCGAATCAGC |
| 2 | GTGTGTTGTGATCATTGTTCCG |
| 3 | CTAATATGTATGAGGTAAGCAATATGTGC |
| 4 | GTTTAACTTACCAACCTTTGCCAG |
| 5 | CTCATAAGGAACTGTGAATTCTTTGATTC |
| 6 | GAACTTCATGAACTGGATACAAGTTC |

**Supplementary Tab. S2. Genes encoding key enzymes involved in the heterotrophic metabolism of *Blautia luti* and genes encoding HydM ortholougs in other *Blautia* species.**

| **Enzyme** | **Locus-Tag** |
| --- | --- |
|  |  |
| Pyruvate-formate lyase | GKZ57_RS04050  GKZ57_RS07555 |
| Pyruvate-ferredoxin oxidoreductase | GKZ57_RS04190 |
| Lactate dehydrogenase | GKZ57_RS06205  GKZ57_RS08460 |
| Transhydrogenase *nfnA* | GKZ57_RS04775 |
| Transhydrogenase *nfnB* | GKZ57_RS04770 |
| Fumarate reductase subunit B | GKZ57_RS08530 |
| Fumarate reductase subunit A | GKZ57_RS08525 |
| PEP carboxykinase | GKZ57_RS08350 |
| Carboxylating malate dehydrogenase | GKZ57_RS13215  GKZ57_RS08130 |
| Hydrogenase *hydM* | GKZ57_RS08550 |
| Electron-bifurcating hydrognease *hydA* | GKZ57_RS08565 |
| Electron-bifurcating hydrognease *hydB* | GKZ57_RS08560 |
| Electron-bifurcating hydrognease *hydC* | GKZ57_RS08555 |
|  |  |
| **Accesion numbers of *hydM* of different *Blautia* species** | |
|  |  |
| *B. obeum* | C4886_RS15775 |
| *B. hydrogenotrophica* | ACMIKQ_RS07960 |
| *B. coccoides* | ACK2JM_RS02005 |
| *B. caecimuris* | U0N64_00960 |
| *B. producta* | E5259_RS20065 |
| *B. massiliensis* | JTJ22_RS15065 |

|  |  |
| --- | --- |
